# Phase I Study of Cetuximab, Irinotecan, and Vandetanib (ZD6474) as Therapy for Patients with Previously Treated Metastastic Colorectal Cancer

**DOI:** 10.1371/journal.pone.0038231

**Published:** 2012-06-12

**Authors:** Jeffrey A. Meyerhardt, Marek Ancukiewicz, Thomas A. Abrams, Deborah Schrag, Peter C. Enzinger, Jennifer A. Chan, Matthew H. Kulke, Brian M. Wolpin, Michael Goldstein, Lawrence Blaszkowsky, Andrew X. Zhu, Meaghan Elliott, Eileen Regan, Rakesh K. Jain, Dan G. Duda

**Affiliations:** 1 Department of Medical Oncology, Dana-Farber Cancer Institute, Boston, Massachusetts, United States of America; 2 Steele Laboratory of Tumor Biology, Department of Radiation Oncology, Massachusetts General Hospital, Boston, Massachusetts, United States of America; 3 Beth-Israel Deaconess Medical Center, Boston, Massachusetts, United States of America; 4 Division of Medical Oncology, Massachusetts General Hospital, Boston, Massachusetts, United States of America; Ohio State University, United States of America

## Abstract

**Background:**

To determine the maximum tolerated dose (MTD) and safety, and explore efficacy and biomarkers of vandetanib with cetuximab and irinotecan in second-line metastatic colorectal cancer.

**Methods:**

Vandetanib (an orally bioavailable VEGFR-2 and EGFR tyrosine kinases inhibitor) was combined at 100 mg, 200 mg, or 300 mg daily with standard dosed cetuximab and irinotecan (3+3 dose-escalation design). Ten patients were treated at the MTD and plasma angiogenesis biomarkers (VEGF, PlGF, bFGF, sVEGFR1, sVEGFR2, IL-1β, IL-6, IL-8, TNF-α, SDF1α) were measured before and after treatment.

**Results:**

Twenty-seven patients were enrolled at 4 dose levels and the MTD. Two dose-limiting toxicities (grade 3 QTc prolongation and diarrhea) were detected at 300 mg of vandetanib with cetuximab and irinotecan resulting in 200 mg being the MTD. Seven percent of patients had a partial response, 59% stable disease and 34% progressed. Median progression-free survival was 3.6 months (95% CI, 3.2–5.6) and median overall survival was 10.5 months (95% CI, 5.1–20.7). Toxicities were fairly manageable with grade 3 or 4 diarrhea being most prominent (30%). Vandetanib and cetuximab treatment induced a sustained increase in plasma PlGF and a transient decrease in plasma sVEGFR1, but no changes in plasma VEGF and sVEGFR2.

**Conclusions:**

Vandetanib can be safely combined with cetuximab and irinotecan for metastatic colorectal cancer. Exploratory biomarker analyses suggest differential effects on certain plasma biomarkers for VEGFR inhibition when combined with EGFR blockade and a potential correlation between baseline sVEGFR1 and response. However, while the primary endpoint was safety, the observed efficacy raises concern for moving forward with this combination.

**Trial Registration:**

Clinicaltrials.gov NCT00436072.

## Introduction

Colorectal cancer is the fourth most common malignancy and second most frequent cause of cancer-related death in the United States, with 141,210 new cases and 49,380 deaths anticipated in 2011. [1] Nineteen percent of patients with colorectal cancer have metastatic disease at the time of diagnosis [2] and nearly 50% of patients who are initially diagnosed with localized disease ultimately develop metastases. [3] While there have been substantive advances in the treatment of metastatic colorectal cancer over the past decade, [4] median survival for these patients remains under 2 years in most trials [5] and less than 10% survive for more than 5 years. New treatment strategies need to be explored.

The two “biologic” therapeutic strategies that have demonstrated activity in metastatic colorectal cancer target the epidermal growth factor receptor (EGFR) and vascular endothelial growth factor (VEGF) both in first and second-line of therapy. [6], [7], [8] However, whereas monoclonal antibodies against EGFR have proven modest efficacy both as monotherapy and in combination with chemotherapy in patients with metastatic disease [9], [10], [11], receptor tyrosine kinase inhibitors (TKIs) of the EGFR, such as erlotinib and gefitinib, do not appear to have appreciable activity against metastatic colorectal cancer as single agents or in combination with cytotoxic chemotherapy. [12], [13], [14], [15], [16] Dual (antibody + TKI) targeting of EGFR has been shown to overcome a major drug resistance mutation in mouse models of EGFR mutant lung cancer. [17] However, whether combined targeting of the extracellular and intracellular domains of EGFR would be more efficacious in metastatic colorectal cancer remains not known. Furthermore, while potential synergistic activity has been hypothesized for combination of EGFR and VEGFR inhibitors [18], [19], [20], [21], previous trials have been inconclusive due to lack of synergy between monoclonal antibodies against VEGF and EGFR and toxicities seen with such drug combinations and chemotherapies. [22], [23], [24].

Vandetanib is an oral multi-targeted antagonist of VEGFR2 and EGFR. [25] In lung cancer, vandetanib was the first TKI with anti-VEGFR2 activity that significantly prolonged progression-free survival when combined with chemotherapy in lung cancer. [26] Thus, combining vandetanib with cetuximab provides an opportunity to explore the effects of inhibiting both the extracellular and intracellular domains of EGFR in cancer cells in conjunction with antiangiogenic/antivascular effects of VEGFR2 inhibition. To date, KRAS mutation status remains the only biomarker used for cetuximab treatment, and there are no validated biomarkers of anti-VEGF therapies. [27] Here, we conducted a multi-center phase I study to assess the safety of combining cetuximab, irinotecan and vandetanib and explore efficacy and biomarkers for the treatment of previously treated metastatic colorectal cancer patients.

## Methods

### Patients

Patients were eligible if they had metastatic colorectal adenocarcinoma and had received 1–2 prior chemotherapy regimens for metastatic disease (prior adjuvant therapy completed within 12 months of enrollment was considered 1 prior regimen). At study onset (February 2007), data on treatment interaction between KRAS mutation status and cetuximab was not known and thus the first 7 patients were not selected by KRAS status; the protocol was amended on July 17, 2008 to restrict to only patients with KRAS wildtype tumors. Patients had to have measurable disease by Response Evaluation Criteria in Solid Tumors Group (RECIST), [28] an Eastern Cooperative Oncology Group (ECOG) performance status 0–2, and adequate hematological, hepatic and renal function. Patients could not have been previously treated with prior EGFR inhibitor (prior irinotecan was permitted). Exclusion criteria included uncontrolled serious medical or psychiatric illness, other malignancy within past 3 years (except limited basal cell or squamous cell carcinoma of the skin or *in situ* cervix carcinoma), inadequately controlled hypertension (blood pressure >160/100 mmHg on antihypertensive medications), clinically significant cardiac event such as myocardial infarction or New York Heart Association classification of heart disease >2 within 3 months of study entry, history of ventricular arrhythmia that was either symptomatic or required treatment, potassium <4.0 mEq/L despite supplementation, serum calcium (ionized or adjusted for albumin) or magnesium out of normal range despite supplementation, previous history of QTc prolongation as a result from other medication that required discontinuation of that medication, congenital long QT syndrome, first degree relative with unexplained sudden death under 40 years of age, presence of left bundle branch block, QTc with Bazett’s correction that is unmeasurable or ≥480 msec on ECG, concomitant medication that may cause QTc prolongation induced Torsades de Pointes or induce CYP3A4 function, lack of physical integrity of the upper gastrointestinal tract or malabsorption syndrome, currently active diarrhea that may affect ability to absorb vandetanib or tolerate potential diarrhea from study drugs, pregnancy or active lactation, and incompletely healed surgical incisions.

Patients were accrued from Brigham and Women’s Hospital, Massachusetts General Hospital and Beth Israel Deaconess Medical Center in Boston. The study was approved by the Dana-Farber/Harvard Cancer Center Institutional Review Board which oversees studies at all three hospitals. All patients signed informed consent.

The protocol for this trial and supporting CONSORT checklist are available as supporting information; see Checklist S1 and Protocol S1.

### Treatment

This was a phase I trial with an expanded maximum tolerated dose (MTD) cohort. Throughout the study, standard dosing of cetuximab was utilized (400 mg/m^2^ loading dose followed by weekly 250 mg/m^2^ doses). Dose level 1 was without irinotecan (vandetanib and cetuximab only); all subsequent dose levels included irinotecan starting on day 15 of therapy at 180 mg/m^2^ intravenously every other week. Dose levels 2, 3, and 4 including oral vandetanib daily at 100 mg, 200 mg and 300 mg, respectively, with cetuximab and irinotecan. Patients were enrolled into each dose level initially in cohorts of 3. No intrapatient dose escalation was permitted. If all 3 patients treated at a dose level were observed during cycle 1 without dose-limiting toxicity, then a new cohort of 3 patients received the next dose level. If 2 of the initial 3 patients experienced a dose-limiting toxicity (DLT), then the previous dose was considered the MTD. If a DLT was observed in one of the initial 3 patients, then 3 additional patients were treated at that dose level. If none of those 3 additional patients experienced a DLT, then the next dose level was administered; otherwise, the previous dose was considered the MTD. Ten additional patients were treated at the MTD. DLTs were defined as specific toxicities observed in the first 28 days of dose level 1 and the first 35 days of dose levels 2–4 (due to the delayed introduction of irinotecan). The DLTs included grade IV hematological toxicity >7 days, fever and neutropenia, grade III diarrhea leading to hospitalization or lasting >48 hours despite aggressive anti-diarrheal medication, grade IV diarrhea despite aggressive anti-diarrheal medication, grade IV vomiting despite optimal antiemetics, grade III or higher nonhematological toxicity (excluding nausea, vomiting, diarrhea, or alopecia) lasting >1 week, grade 4 skin toxicity, grade 3 or greater cardiac toxicities or death from any cause.

**Figure 1:**
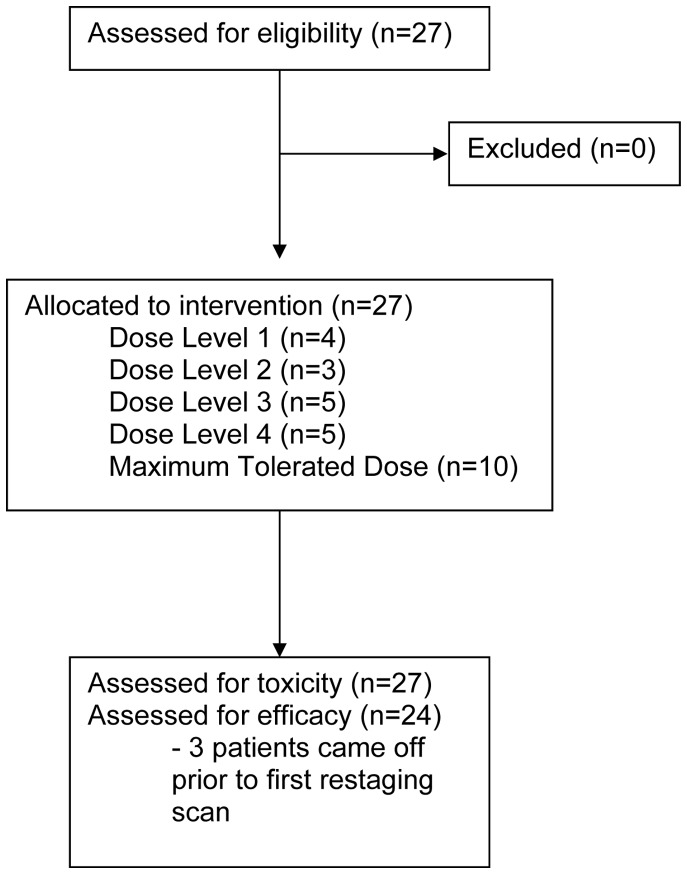
Study CONSORT Flow Diagram.

All toxicities were graded according to the National Cancer Institute (NCI) Common Toxicity Criteria (CTC) version 3.0. Dose reductions for vandetanib depended on starting dosage, with initially 100 mg per day reductions and finally reduction from 100 mg daily to every other day. If further reductions were needed, patient was withdrawn from the study. Due to concerns regarding QTc prolongation with vandetanib, initially frequent EKGs were performed and vandetanib was held for patients with either a single QTc value of 550 msec or greater, single QTc increase of 100 msec or greater from baseline, or two consecutive EKG measurements within 48 hours of one another in which the mean QTc interval from 3 EKGs is greater than 500 but less than 550 msec or the mean QTc interval increase from baseline is greater than 60 but less than 100. Upon treatment hold, efforts were made to replete appropriate electrolytes and treatment resumed once QTc resolved to within 60 msec of baseline at a dose reduction. Further increases of QTc required removal of the patient from the study. Vandetanib was held for grade 3 or 4 cutaneous reactions as well as other grade 3 or 4 toxicities deemed associated with the study medication, with resumption once resolved to grade 1 or less toxicity with a dose reduction. Vandetanib could be held for up to 3 weeks.

Cetuximab and irinotecan dose modifications were consistent with other protocols [9], [29] and their individual package labels.

Treatment was continued until development of progressive disease by RECIST, unacceptable toxicity, withdrawal of consent, intercurrent illness that prevented continuation of therapy, or changes in the patient’s condition that rendered him or her unable to continue study drugs (as judged by the treating clinician).

### Response Evaluation

Baseline tumor measurements by computer tomography were obtained within 28 days before treatment was initiated. Treatment cycles were defined at every 8 weeks. Study visits included toxicity assessment, physical examination, and laboratory studies were conducted weekly during cycle 1 and then every other week for subsequent cycles. Patients were asked to keep a diary of their self-administration of vandetanib as well as record daily side effects; these diaries were reviewed at each study visit. EKGs were obtained at baseline, weeks 1, 2, 4, 8 and then day 1 of every subsequent cycle.

Repeat imaging was required prior to start of each cycle. Evaluation of response, stable disease and disease progression was based on RECIST. [28] Confirmation scans for responders were performed at least 4 weeks after the initial scan documenting the reduction.

**Table 1:** Baseline characteristics (n = 27).

Characteristic	Distribution
Age (years)	
Median	53
Range	31–74
Gender	
Female	13 (48%)
Male	14 (52%)
Race	
Caucasian	24 (89%)
Other	3 (11%)
Baseline ECOG Performance Status	
0	12 (44%)
1–2	15 (56%)
Prior lines of therapy (including prior adjuvant therapy)	
1	15 (56%)
2	10 (37%)
3	2 (7%)
Prior irinotecan-based therapy	15 (56%)
Prior oxaliplatin-based therapy (adjuvant or metastatic or both)	22 (81%)
Site of primary tumor	
Colon	21 (78%)
Rectum	6 (22%)

### Biomarker Studies

Exploratory biomarker studies were conducted in the 10 patients treated at the MTD. Blood samples were collected prior to first dose of any therapy on day 1 then on days 8, 15, 22 of cycle 1 then on day 1 of every subsequent cycle. Circulating angiogenic and inflammatory biomarkers were measured in plasma. Analysis was carried out for circulating VEGF, placental growth factor (PlGF), soluble VEGF receptor 1 (sVEGFR1), basic fibroblast growth factor (bFGF), interleukin (IL)-1β, IL-6, IL-8, tumor necrosis factor α (TNFα) using multiplex enzyme-linked immunosorbent assay (ELISA) plates from Meso-Scale Discovery (Gaithersburg, MD). sVEGFR2 and stromal cell–derived factor 1α (SDF1α) were similarly analyzed using ELISA plates from R&D System (Minneapolis, MN). Every sample was run in duplicate.

**Table 2:** Adverse Events (based on worse toxicity by patient).

	Dose Level 1N = 4		Dose Level 2N = 3		Dose Level (MTD)N = 15		Dose Level 4N = 5	
Toxicity	Grade 1/2n (%)	Grade 3/4n (%)	Grade 1/2n (%)	Grade 3/4n (%)	Grade 1/2n (%)	Grade 3/4n (%)	Grade 1/2n (%)	Grade 3/4n (%)
Rash	4 (100%)	–	3 (100%)	–	13 (87%)	2 (13%)	4 (80%)	1 (20%)
Electrolyte changes*	4 (100%)	–	2 (67%)	1 (33%)	9 (69%)	3 (20%)	3 (60%)	1 (20%)
Fatigue	4 (100%)	–	3 (100%)	–	10 (67%)	–	4 (80%)	–
Dry skin/Pruritus	1 (25%)	–	1 (33%)	–	6 (40%)	1 (7%)	4 (80%)	–
Diarrhea	1 (25%)	–	2 (67%)	1 (33%)	11 (73%)	4 (27%)	2 (40%)	3 (60%)
Nausea	1 (25%)	–	1 (33%)	–	6 (40%)	–	3 (60%)	–
Emesis	1 (25%)	–	2 (67%)	–	4 (27%)	–	3 (60%)	–
Anorexia	2 (50%)	–	1 (33%)	–	3 (20%)	–	4 (80%)	–
Liver function changes	1 (25%)	–	2 (67%)	–	6 (40%)	1 (7%)	3 (60%)	1 (20%)
Neutropenia	–	–	1 (33%)	1 (33%)	–	1 (7%)	–	1 (20%)
Lymphopenia	1 (25)	–	3 (100%)	–	4 (27%)	–	2 (40%)	–
Anemia	–	–	1 (33%)	–	3 (20%)	–	1 (20%)	–
Thrombocytopenia	–	–	–	–	2 (13%)	–	1 (20%)	–
Headache	1 (25%)	–	3 (100%)	–	2 (13%)	–	2 (40%)	–
Nail changes	–	–	1 (33%)	–	4 (27%)	–	2 (40%)	–
Dehydration	–	–	–	–	3 (20%)	–	2 (40%)	–
Constipation	–	–	1 (33%)	–	3 (20%)	–	2 (40%)	–
Abdominal pain	2	–	1 (33%)	1 (33%)	1 (7%)	–	2 (40%)	–
Other pain	1 (25%)	–	1 (33%)	1 (33%)	1 (7%)	–	2 (40%)	–
Shortness of breath	2 (50%)	–	–	1 (33%)	2 (13%)	–	–	–
Fever without neutropenia	1 (25%)	–	1 (33%)	–	2 (13%)	–	–	–
Creatinine elevation			1 (33%)	–	–	1 (7%)	1 (20%)	–
**Toxicity**	**Dose Level 1** **N = 4**		**Dose Level 2** **N = 3**		**Dose Level 3/MTD** **N = 15**		**Dose Level 4** **N = 5**	
Ocular irritation	1 (25%)	–	–	–	1 (7%)	–	–	–
QTc prolongation	–	–	–	–	–	–	–	1 (20%)
Hypertension	–	–	–	–	–	1 (7%)	1 (20%)	–
Proteinuria	–	–	–	–	2 (13%)	–	1 (20%)	–
Neuropathy	–	–	–	–	2 (13%)	–	–	–
Alopecia	–	–	–	–	2 (13%)	1 (7%)	–	–
Any grade 3 or 4	–	–	–	2 (67%)	–	8 (53%)	–	5 (100%)

* includes magnesium, potassium, sodium and calcium changes.

### Statistical Analysis

The primary endpoint of this study was to determine the tolerability and maximum tolerated dose of combining vandetanib, cetuximab and irinotecan in patients with metastatic colorectal cancer refractory to prior cytotoxic chemotherapy. Secondary endpoints were determinations of response rate, progression-free survival and overall survival of this combination. Responses were determined by RECIST with an intention-to-treat analysis. [28] Progression-free survival (PFS) was defined as the time between study enrollment and progression of disease or death. Overall survival (OS) was defined as the time between study enrollment and death and estimated by the Kaplan-Meier method. [30].

For circulating biomarkers, data were reported as median and interquartile range and the P-values were determined using the paired exact Wilcoxon test. We adjusted P values for multiple comparisons over time, using the false discovery rate control method of Genovese and colleagues [31], with weights proportional to the square root of the number of data. Kendall’s nonparametric coefficients of correlation τ_β_ and the two-sided Kendall’s test were used to quantify the correlation of biomarkers with tumor relative size change and RECIST criteria and to test τ_β_ = 0.

**Figure 2:**
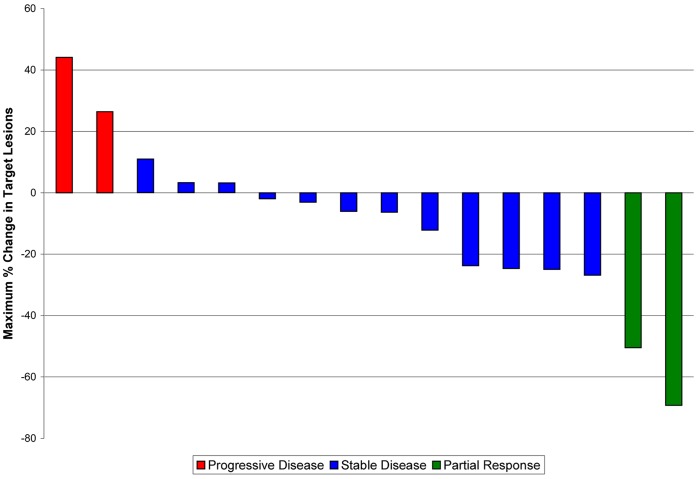
Waterfall Plot. Best response analysis after vandetanib with cetuximab and irinotecan limited to patients with confirmed KRAS wild-type metastatic colorectal cancer and at least 1 restaging imaging scan.

**Figure 3:**
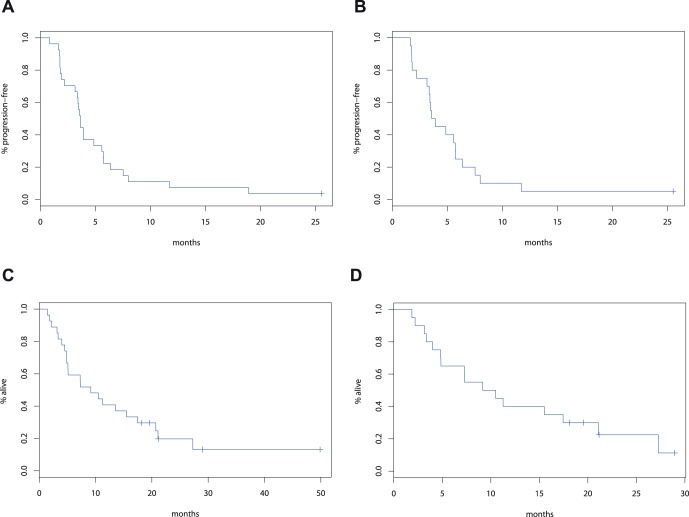
Kaplan-Meier Curves of Progression-Free Survival (PFS) and Overall Survival (OS) Distributions after Vandetanib with Cetuximab and Irinotecan in Metastatic Colorectal Cancer Patients. **A**, PFS distribution for all patients (n = 27). **B**, PFS distribution for patients with confirmed KRAS wild-type tumors (n = 20). **C**, PFS distribution for all patients (n = 27). **D**, PFS distribution for patients with confirmed KRAS wild-type tumors (n = 20).

## Results

### Patient Characteristics

Twenty-seven patients enrolled into this phase I study of vandetanib in combination with irinotecan and cetuximab from March 2007 to March 2010 (**Figure 1**). Baseline characteristics of these patients are summarized in **Table 1**. The median age of the patients was 53, 52% were male, and 11% were race other than Caucasian. Over half of all patients had a baseline ECOG performance status of 1 or 2 and 44% had received 2 or more prior therapies for metastatic colorectal cancer.

**Table 3:** Pre-treatment values and fold-changes in plasma biomarkers after treatment with vandetanib and cetuximab (days 8 and 15) and with vandetanib, cetuximab and irinotecan (day 21, cycle 3 and cycle 5) in metastatic colorectal cancer patients.

Biomarker	Pre-treatment	Day 8	Day 15	Day 21	Cycle 3	Cycle 5
**VEGF**	878 pg/ml [485,1559] (n = 8)	0.92 [0.86,1.05](n = 7)	0.86 [0.81,0.97](n = 8)	0.67 [0.57,0.74](n = 8)	0.41 [0.23,0.67](n = 7)	1.27 [0.86,1.05](n = 4)
P		0.69	0.11	0.016	0.47	0.88
Adjusted P		0.86	0.18	0.078	0.12	0.88
**PlGF**	37 pg/ml [29], [44] (n = 8)	**1.36 [1.32, 1.46]** **(n = 7)**	**1.34 [1.09, 1.50]** **(n = 8)**	**1.60 [1.44, 1.87]** **(n = 8)**	1.64 [1.06, 1.68](n = 7)	1.44 [1.24, 1.81](n = 4)
P		**0.016**	**0.016**	**0.0078**	0.078	0.13
Adjusted P		**0.026**	**0.026**	**0.026**	0.098	0.13
**sVEGFR1**	470 pg/ml [277,677] (n = 8)	0.70 [0.65,1.04](n = 7)	**0.49 [0.41,0.57]** **(n = 8)**	0.60 [0.17,0.83](n = 8)	0.85 [0.35,1.08](n = 7)	0.29 [0.21,0.47](n = 4)
P		0.22	**0.0078**	0.039	0.30	0.13
Adjusted P		0.27	**0.039**	0.098	0.30	0.21
**sVEGFR2**	5,040 pg/ml [4,490, 7,030] (n = 8)	1.02 [0.99,1.15](n = 7)	0.95 [0.91,1.12](n = 8)	0.93 [0.91,1.04](n = 8)	0.93 [0.83,1.04](n = 7)	0.84 [0.78,0.90](n = 4)
P		0.37	0.95	0.31	0.30	0.25
Adjusted P		0.47	0.95	0.47	0.47	0.47
**bFGF**	40.7 pg/ml [24.2,151.9] (n = 8)	1.09 [0.70, 4.06](n = 7)	1.70 [0.78, 2.20] (n = 8)	1.35 [0.66, 5.07](n = 8)	3.66 [0.69, 6.53](n = 7)	3.12 [1.98, 6.33](n = 4)
P		0.81	0.64	0.46	0.47	0.25
Adjusted P		0.81	0.80	0.78	0.78	0.78
**SDF1α**	2,532 pg/ml [2,380, 3,304] (n = 8)	1.12 [0.97, 1.25](n = 7)	1.07 [0.89, 1.24](n = 8)	1.05 [0.94, 1.28](n = 8)	1.18 [0.86, 1.23](n = 7)	0.99 [0.81, 1.25](n = 4)
P		0.58	0.95	0.55	0.69	1.0
Adjusted P		1.0	1.0	1.0	1.0	1.0
**IL-1β**	0.60 pg/ml [0.40,0.85] (n = 8)	1.00 [0.83, 1.82](n = 7)	1.05 [0.62, 1.52] (n = 8)	0.96 [0.56, 1.62](n = 8)	1.43 [0.92, 1.93](n = 7)	0.67 [0.46, 0.99](n = 4)
P		0.44	0.81	0.95	0.30	0.38
Adjusted P		0.73	0.95	0.95	0.73	0.73
**IL-6**	3.70 pg/ml [2.70,5.32] (n = 8)	1.19 [0.84, 1.68] (n = 7)	1.60 [1.06, 2.34](n = 8)	1.78 [1.36, 3.25](n = 8)	2.07 [1. 23, 3.80](n = 7)	0.99 [0.71, 1.25](n = 4)
P		0.38	0.20	0.25	0.30	0.88
Adjusted P		0.47	0.47	0.47	0.47	0.88
**IL-8**	15.82 pg/ml [8.65,31.49] (n = 8)	0.81 [0.38,0.86](n = 7)	0.80 [0.58,1.02](n = 8)	0.99 [0.70,1.28](n = 8)	0.81 [0.62,1.16](n = 7)	0.47 [0.40, 1.03](n = 4)
P		0.22	0.31	0.95	0.69	0.63
Adjusted P		0.27	0.86	0.95	0.86	0.86
**TNF-α**	11.9 [10.2,14.4] (n = 8)	1.14 [0.95, 1.47](n = 7)	1.11 [0.90, 1.38](n = 8)	0.95 [0.64, 1.23](n = 8)	1.04 [0.84, 1.39](n = 7)	0.72 [0.63, 0.95](n = 4)
P		0.47	0.38	0.64	0.69	0.38
Adjusted P		0.69	0.69	0.69	0.69	0.69

Data are shown as medians and interquartile ranges (in square brackets) compared to baseline levels. *P*-values are from the exact paired Wilcoxon test, before and after adjustment for multiple comparisons over time using the method of Genovese at al. VEGF, vascular endothelial growth factor; bFGF, basic fibroblast growth factor; PlGF, placental growth factor; sVEGFR-1, soluble VEGF receptor-1; sVEGFR-2, soluble VEGF receptor-2; SDF-1α, stromal cell-derived factor-1-alpha; IL-6, interleukin-6; IL-8, interleukin-8; TNF-α, tumor necrosis factor-alpha.

### Determination of the Maximum Tolerated Dose

Four patients were enrolled at dose level 1 (vandetanib 100 mg per day with cetuximab) without a DLT (one patient was added due to symptomatic disease progression prior to 28 day required period for DLT detection in one of the first 3 patients). Dose levels 2 and 3 added irinotecan to cetuximab and vandetanib (100 mg per day and 200 mg per day, respectively) and no DLTs were detected. Three patients were enrolled at dose level 2 and 5 were enrolled at dose level 3 because 1 patient had symptomatic progression within the DLT monitoring period and 2 patients were consented simultaneously. At dose level 4 (i.e., vandetanib 300 mg per day with cetuximab and irinotecan), one of the initial 3 patients experienced a DLT (grade 3 QTc prolongation). The dose level 4 cohort was expanded to an additional 3 patients however the second patient experienced a DLT (grade 3 diarrhea). Therefore, dose level 4 was considered too toxic and MTD was determined to be vandetanib 200 mg per day, irinotecan 180 mg/m^2^ intravenously every other week and cetuximab 400 mg/m^2^ loading dose followed by weekly cetuximab 250 mg/m^2^. Ten additional patients were treated at the MTD.

### Toxicity

Twenty-seven patients were evaluated for treatment-associated toxicities, by dose level of treatment (**Table 2**). While dose level 4 was discontinued due to two patients experiencing protocol defined DLTs, overall toxicities appeared greater by percentage compared to the other dose levels as well. In considering the entire cohort, all patients developed some level of a rash, with 11% being classified as grade 3. Electrolyte changes were also very common (85% of patients) though only 5 patients (19%) experienced grade 3 or 4 abnormalities, 4 with hypomagnesium and 1 with hypokalemia. Fatigue was seen in 78% of patients, though all were classified as grade 1 or 2.

Any grade 3 or 4 toxicity was observed in 59% of patients, with all patients in dose level 4 experiencing at least one. Besides electrolyte abnormalities and rash, 8 patients (30%) had grade 3 or 4 diarrhea, 3 patients (11%) had grade 3 neutropenia, 1 patient had grade 3 and 1 patient had grade 4 liver function abnormalities. Other grade 3 toxicities experienced by a single patient included pruritus, abdominal pain, shortness of breath, creatinine elevation, ocular irritation, QTc prolongation, hypertension, and alopecia.

### Efficacy and Duration of Treatment

Among the 27 patients enrolled in this phase I trial of vandetanib, cetuximab and irinotecan, 24 patients were evaluable for radiographic response (3 were removed from study due to toxicity prior to restaging scans). Amongst the 24 patients, 12.5% had a partial response, 62.5% had stable disease and 25% had progressive disease as best response (**Figure 2**). The median PFS for the entire cohort of 27 patients was 3.6 months (95% confidence interval [CI], 2.2–5.6) and median OS was 9.2 months (95% CI, 4.8–17.4) (**Figure 3**). When considering only the 20 patients known to be KRAS wild type, median PFS was 3.7 months (95% CI, 2.2–5.7) and median OS 9.8 months (95% CI, 4.0–21.0). When analyses are limited to only the 15 patients treated at the MTD (including those treated at those doses during dose determination phase), the median PFS was 5.6 months (95% CI, 1.8–6.4) and median OS 15.5 months (95% CI, 3.2–27.3).

Using intent-to-treat analyses, 15 patients (56%) came off study due to progression of disease. Ten patients (37%) stopped therapy due to toxicity. One patient who experienced stable disease as best response underwent curative-intent surgery for liver metastases. Finally, one patient withdrew consent due to need for Achilles tendon surgery.

### Exploratory Analyses of Plasma Biomarkers

Vandetanib and cetuximab treatment increased the plasma concentration of plasma PlGF and decreased sVEGFR1 (p<0.05) (Table 3). The increase in plasma PlGF was maintained after irinotecan was added to vandetanib and cetuximab. In contrast, plasma sVEGFR2, VEGF, bFGF, SDF1α, IL-1β, IL-6, IL-8 and TNF- α were not significantly changed in this cohort after vandetanib and cetuximab nor after addition of irinotecan in these patients. Of all biomarkers, baseline (pre-treatment) sVEGFR1 correlated inversely with response assessed by RECIST (p<0.05).

## Discussion

In this phase I study of patients with previously treated metastatic colorectal cancer, we determined that oral vandetanib at 200 mg daily could be safely combined with weekly cetuximab and every other week irinotecan. As defined by the protocol a priori, diarrhea and QTc interval prolongation were DLTs at a higher dose of vandetanib. However, the activity of the regimen at the MTD was rather modest and not appreciably different from historical data for cetuximab and irinotecan in KRAS wild-type patients.

The landscape of treatment for colorectal cancer had changed at a rapid pace since 1998 and 2006. Prior to 1998, the only drug approved was 5-fluorouracil. However, in the span of less than a decade, two additional cytotoxic chemotherapies (irinotecan and oxaliplatin), two inhibitors of EGFR (cetuximab and panitunumab) and one monoclonal antibody against VEGF (bevacizumab) have demonstrated efficacy in randomized phase III trials and are being routinely incorporated into regimens to treat metastatic colorectal cancer. Despite improvements in median survival from less than 1 year to greater than 2 years in this time period, most patients with metastatic colorectal cancer will still eventually die from their disease. Many efforts are currently underway to target new pathways important to the growth and metastatic potential of colorectal cancer.

Another potential strategy to improve survival from colorectal cancer is to further capitalize on the pathways that have had some therapeutic potential in this disease, EGFR and VEGFR. Cetuximab and panitumumab target the extracellular domain of EGFR, blocking ligand binding. Preclinical experiments support combining EGFR monoclonal antibodies with oral inhibitors of EGFR tyrosine kinase, with synergistic effects on proliferation and induction of apoptosis as well as enhancement of phosphorylation inhibition of downstream effector molecules (e.g., MAPK and AKT). [32], [33] Vandetanib is receptor tyrosine kinase inhibitor with inhibitory activity against EGFR (IC_50_ = 500 nM) tyrosine kinase. [34], [35] In addition, vandetanib inhibits vascular endothelial growth factor receptor-2 (VEGFR-2) (VEGFR-2: IC_50_ = 40 nM) and Rearranged during Transfection (RET: IC_50_ = 100 nM) tyrosine kinase activity. [36], [37] Vandetanib has been recently approved for advanced medullary thyroid cancer, a disease driven by activating mutations in the RET proto-oncogene [38], but has also shown activity with chemotherapy in lung cancer, most likely due to EGFR and potentially VEGFR2 inhibition. [26] This supported the rationale for testing vandetanib with cetuximab as a unique strategy to dual targeting of EGFR extracellular and intracellular domains as well as combined EGFR and VEGFR2 inhibition.

This phase I trial of vandetanib, cetuximab and irinotecan was initiated prior to the discovery of KRAS status as marker of cetuximab activity. [39] The protocol was amended after the first seven patients to limit to those most likely to benefit from cetuximab and irinotecan. The anticipated benefit of cetuximab and irinotecan in second-line metastatic colorectal cancer is median PFS of 5–5.5 months and median OS of 11 months, based on retrospective data from a large cohort of 448 patients with previously treated, KRAS wildtype, metastatic colorectal cancer treated with cetuximab plus chemotherapy. [40] While the sample size in this current trial limits tight confidence intervals around the efficacy endpoints, there does not appear to be appreciable improvement in PFS or OS with this combination.

The lack of efficacy for this strategy raises two issues that have become increasingly apparent in other studies of VEGF and EGFR inhibitors. First, unlike antibodies, using TKIs for targeting EGFR (e.g., gefitinib or erlotinib) or VEGFR (e.g., vatalanib, sunitinib, sorafenib and cediranib) has shown disappointing results in trials of TKI combined with chemotherapy for metastatic colorectal cancer; [12], [13], [14], [23], [41], [42], [43], [44], [45], [46], [47] similarly, all phase III trials of single agent TKI have failed with the exception of one trial utilizing regorafenib in latter line therapy. [15], [16], [45], [48] The biological rationale of the limited activity of these small molecular inhibitors compared to monoclonal antibodies against the same receptor is not clear. Second, combination trials of cetuximab or panitumumab with bevacizumab have also led to disappointing and concerning results, at least when tested in first-line therapy. [22], [24] Our results using an antibody and a TKI for EGFR and VEGF inhibition supports the lack of efficacy of dual targeting of these pathways in KRAS wild-type metastatic colorectal cancer as opposed to an unknown interaction between monoclonal antibodies.

These underwhelming data notwithstanding, there is an increasing realization that targeted therapies, as well as cytotoxic treatments, will likely only benefit subgroups of patients with a given cancer. Molecular markers are hoped to provide a means to better assess upfront to choose a therapy for a particular patient or to allow for a very early assessment of whether a treatment has potential to benefit. To date, *KRAS* status is the only predictive biomarker that defines the utility of treatment choice in colorectal cancer: mutant *KRAS* patients are excluded from cetuximab treatment. [39] In this trial, we explored plasma markers of angiogenesis and inflammation as markers of activity as well as assessments of whether the therapy was impacted on the assumed molecular process. Consistent with the anti-VEGF activity of vandetanib–and in agreement with data from trials of other VEGF inhibitors in colorectal carcinoma patients (e.g., bevacizumab)–treatment increased the plasma concentration of plasma PlGF and decreased sVEGFR1. [27], [49] The probability of a response increased significantly with lower plasma levels of sVEGFR1 at baseline. Of note, this inverse correlation between pretreatment sVEGFR1 (an endogenous inhibitor of VEGF and PlGF) and outcome has been previously seen in patients with rectal cancer after bevacizumab and chemoradiation [27], [50] as well as after bevacizumab with chemotherapy in breast cancer and cediranib in hepatocellular carcinoma (Sara Tolaney and Andrew Zhu, personal communication). Plasma sVEGFR-2 concentration has been previously proposed as a “pharmacodynamic biomarker” for multiple agents with anti-VEGFR-2 TKI activity when used as monotherapy. [27] Surprisingly, vandetanib did not decrease plasma sVEGFR2 in this cohort. This result may be explained by the relatively weak anti-VEGFR-2 TKI activity of vandetanib [51], [52] or by its use in combination with cetuximab and chemotherapy. Similarly, most anti-VEGF agents increase plasma VEGF levels, [27] including vandetanib in lung cancer patients, [51] while EGFR inhibition is thought to decrease VEGF expression by cancer cells. [53] In this trial, we found a trend toward decreased plasma VEGF after vandetanib, cetuximab and irinotecan treatment. While exploratory, these results suggest that circulating sVEGR1 should be further tested as a predictive biomarker candidate and PlGF should be further tested as a selective and specific pharmacodynamic biomarker candidate for other anti-VEGF therapies. On the other hand, the level of circulating inflammatory cytokines did not change after treatment. Taken together, the biomarker kinetics suggest that despite minimal anti-tumor activity, vandetanib and cetuximab may adequately suppress target (EGFR and VEGFR2) activity in metastatic colorectal cancer.

In conclusion, vandetanib can be safely combined with cetuximab and irinotecan. However, while the primary objective of the study was evaluation of safety of the combination, there is no apparent increase in efficacy of this combination compared to historic data in previously treated metastatic colorectal cancer patients. [6], [54] Despite the apparent lack of improved efficacy, our plasma biomarker data suggest that the anticipated targets may have been impacted, and could potentially benefit a subset of patients. This suggests that future studies should examine specific mechanism escape for EGFR and VEGFR inhibition to design biology-driven approaches for improved therapy in metastatic colorectal cancer.

## Supporting Information

Checklist S1 **CONSORT Checklist.**
(DOC)

Protocol S1 **Trial Protocol.**
(PDF)
