# Supplementary material for: Phase I Study of Cetuximab, Irinotecan, and Vandetanib (ZD6474) as Therapy for Patients with Previously Treated Metastastic Colorectal Cancer
Source: PLoS One. 2012 Jun 12;7(6):e38231. doi: 10.1371/journal.pone.0038231 (PMC3373492; doi:10.1371/journal.pone.0038231)
Supplement: Checklist S1 — CONSORT Checklist. (DOC) [file pone.0038231.s001.doc]

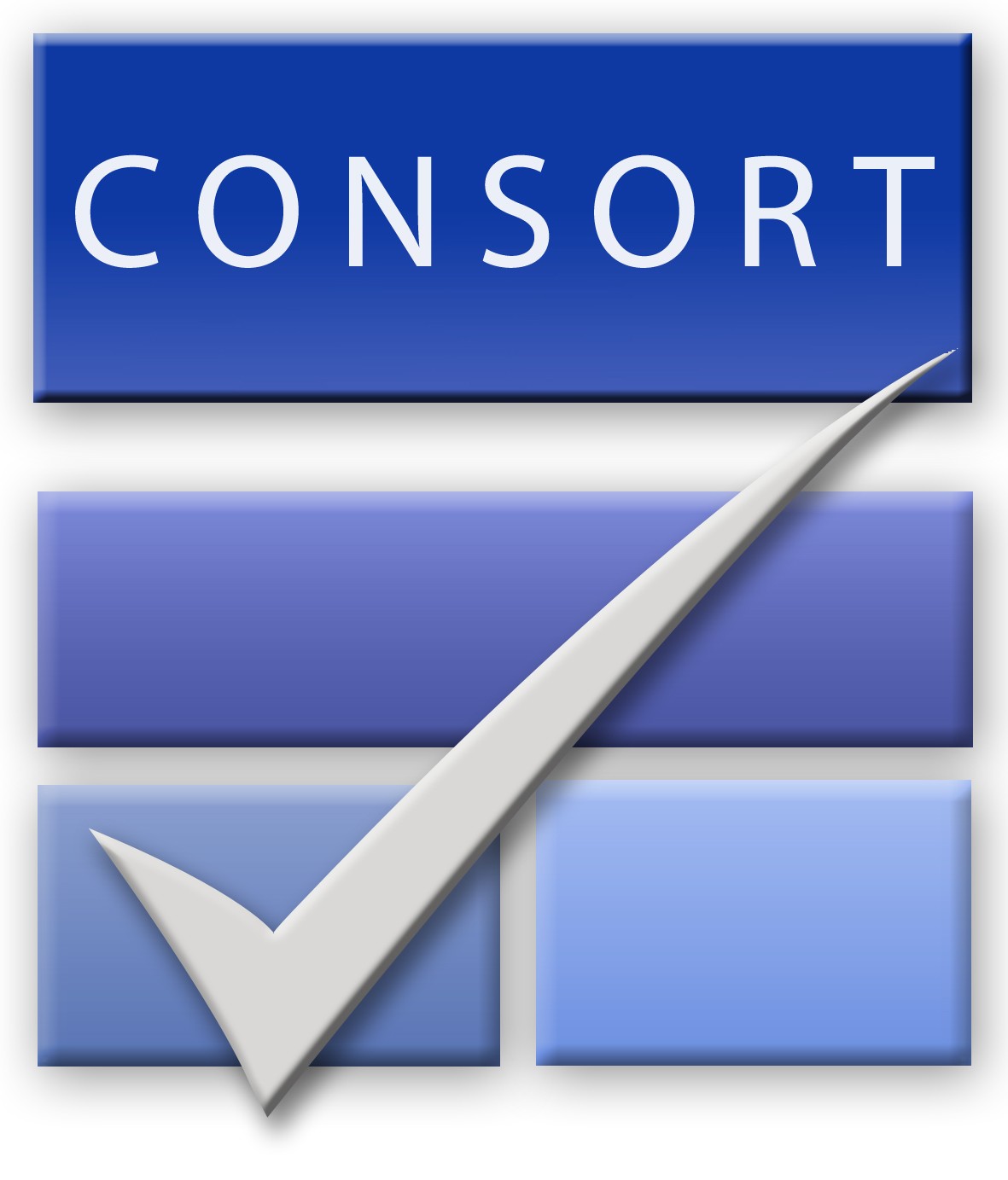
CONSORT checklist for Phase I study of cetuximab, irinotecan, and vandetanib (ZD6474) as therapy for patients with previously treated metastastic colorectal cancer *

| Section/Topic | Item No | Checklist item | Reported in section |
| --- | --- | --- | --- |
| Title and abstract | | | |
|  | 1a | Identification as a randomised trial in the title | Not Applicable |
|  | 1b | Structured summary of trial design, methods, results, and conclusions (for specific guidance see CONSORT for abstracts) | Abstract |
| Introduction | | | |
| Background and objectives | 2a | Scientific background and explanation of rationale | Introduction |
|  | 2b | Specific objectives or hypotheses | Introduction |
| Methods | | | |
| Trial design | 3a | Description of trial design (such as parallel, factorial) including allocation ratio | Patients in Patients and Methods |
|  | 3b | Important changes to methods after trial commencement (such as eligibility criteria), with reasons | Patients in Patients and Methods |
| Participants | 4a | Eligibility criteria for participants | Patients in Patients and Methods |
|  | 4b | Settings and locations where the data were collected | Patients in Patients and Methods |
| Interventions | 5 | The interventions for each group with sufficient details to allow replication, including how and when they were actually administered | Treatments in Patients and Methods |
| Outcomes | 6a | Completely defined pre-specified primary and secondary outcome measures, including how and when they were assessed | Response evaluation in Patients and Methods |
|  | 6b | Any changes to trial outcomes after the trial commenced, with reasons | Not Applicable |
| Sample size | 7a | How sample size was determined | Protocol |
|  | 7b | When applicable, explanation of any interim analyses and stopping guidelines | Not Applicable |
| Randomisation: |  |  |  |
| Sequence generation | 8a | Method used to generate the random allocation sequence | Not Applicable |
|  | 8b | Type of randomisation; details of any restriction (such as blocking and block size) | Not Applicable |
| Allocation concealment mechanism | 9 | Mechanism used to implement the random allocation sequence (such as sequentially numbered containers), describing any steps taken to conceal the sequence until interventions were assigned | Not Applicable |
| Implementation | 10 | Who generated the random allocation sequence, who enrolled participants, and who assigned participants to interventions | Not Applicable |
| Blinding | 11a | If done, who was blinded after assignment to interventions (for example, participants, care providers, those assessing outcomes) and how | Not Applicable |
|  | 11b | If relevant, description of the similarity of interventions | Not Applicable |
| Statistical methods | 12a | Statistical methods used to compare groups for primary and secondary outcomes | Statistical analysis evaluation in Patients and Methods |
|  | 12b | Methods for additional analyses, such as subgroup analyses and adjusted analyses | Statistical analysis evaluation in Patients and Methods |
| Results | | | |
| Participant flow (a diagram is strongly recommended) | 13a | For each group, the numbers of participants who were randomly assigned, received intended treatment, and were analysed for the primary outcome | Patient characteristics |
|  | 13b | For each group, losses and exclusions after randomisation, together with reasons | CONSORT figure |
| Recruitment | 14a | Dates defining the periods of recruitment and follow-up | Patient characteristics |
|  | 14b | Why the trial ended or was stopped | Efficacy and duration of treatment |
| Baseline data | 15 | A table showing baseline demographic and clinical characteristics for each group | Table 1 |
| Numbers analysed | 16 | For each group, number of participants (denominator) included in each analysis and whether the analysis was by original assigned groups | Results |
| Outcomes and estimation | 17a | For each primary and secondary outcome, results for each group, and the estimated effect size and its precision (such as 95% confidence interval) | Efficacy and duration of treatment |
|  | 17b | For binary outcomes, presentation of both absolute and relative effect sizes is recommended | Efficacy and duration of treatment |
| Ancillary analyses | 18 | Results of any other analyses performed, including subgroup analyses and adjusted analyses, distinguishing pre-specified from exploratory | Exploratory Analyses of Plasma Biomarkers |
| Harms | 19 | All important harms or unintended effects in each group (for specific guidance see CONSORT for harms) | Determination of the Maximum Tolerated Dose and Toxicity |
| Discussion | | | |
| Limitations | 20 | Trial limitations, addressing sources of potential bias, imprecision, and, if relevant, multiplicity of analyses | Discussion |
| Generalisability | 21 | Generalisability (external validity, applicability) of the trial findings | Discussion |
| Interpretation | 22 | Interpretation consistent with results, balancing benefits and harms, and considering other relevant evidence | Discussion |
| Other information | | |  |
| Registration | 23 | Registration number and name of trial registry | Abstract (end) |
| Protocol | 24 | Where the full trial protocol can be accessed, if available | Supporting Information |
| Funding | 25 | Sources of funding and other support (such as supply of drugs), role of funders | Acknowledgement |

*We strongly recommend reading this statement in conjunction with the CONSORT 2010 Explanation and Elaboration for important clarifications on all the items. If relevant, we also recommend reading CONSORT extensions for cluster randomised trials, non-inferiority and equivalence trials, non-pharmacological treatments, herbal interventions, and pragmatic trials. Additional extensions are forthcoming: for those and for up to date references relevant to this checklist, see [www.consort-statement.org](http://www.consort-statement.org/).
