# Supplementary material for: Phase I Study of Cetuximab, Irinotecan, and Vandetanib (ZD6474) as Therapy for Patients with Previously Treated Metastastic Colorectal Cancer
Source: PLoS One. 2012 Jun 12;7(6):e38231. doi: 10.1371/journal.pone.0038231 (PMC3373492; doi:10.1371/journal.pone.0038231)
Supplement: Protocol S1 — Trial Protocol. (PDF) [file pone.0038231.s002.pdf]

ON HOLD

ALERT PAGE

PROTOCOL **06-177**

ACCRUAL TO THIS TRIAL IS TEMPORARILY ON HOLD AT BIDMC

Date Posted: October 21, 2010

No new subjects may be enrolled in the study as described above. Any questions regarding this closure should be directed to the study's Principal Investigator

Date Submitted: 03/10/11
*Date Posted: **04/11/11***

Alert Page

DF/HCC Protocol #: 06-177

Dose Escalation Table		
**Effective Date**	**Dose Level / Cohort #**	**Dose (insert units)**
02/12/07	1	100 mg ZD6474,<br>250 mg/m2 Cetuximab
02/22/08	2	100 mg ZD6474,<br>250 mg/m2 Cetuximab,<br>180 mg/m2 Irinotecan
07/22/08	3	200 mg ZD6474,<br>250 mg/m2 Cetuximab,<br>180 mg/m2 Irinotecan
**03/26/2009**	**4**	**300 mg ZD6474<br>250 mg/m2 Cetuximab,<br>180 mg/m2 Irinotecan**

Note: The row for the current dose level is **highlighted and bolded**.

Protocol Clarifications (non-drug related e.g. eligibility criteria, study assessments)

Protocol Section 4.2: Patients must meet eligibility criteria of Potassium $\geq$ 4. Once enrolled, there is no need to withhold ZD6474 if the K is 3.0-4.0. As with other toxicities, the cut-off for holding the dose would be CTCAE grade 3 toxicity (or K <3.0). The patient should continue to receive K supplementation.

PROTOCOL **06-177**

Closed to New Accrual

Closure Effective Date: May 18, 2010

No new subjects may be enrolled in the study as described above. Any questions regarding this closure should be directed to the study's Principal Investigator

DANA-FARBER CANCER INSTITUTE

Office for Human Research Studies

DANA-FARBER / HARVARD CANCER CENTER

Protocol Front Sheet

DFCI Protocol No.: 06-177

1. PROTOCOL TITLE AND VERSION

Title: Phase I ZD6474/CETUXIMAB/IRINOTECAN IN MET COLORECTAL

Protocol Version No./ Date: 03/10/2011 **Sponsor Study Number:** RUSZACT0027

2. DF/HCC STUDY CONTACT INFORMATION

Study Contact for Questions: Meaghan Elliott **Email:** Meaghan_Elliott@dfci.harvard.edu **Phone:** 617-632-6353

OVERALL AND SITE RESPONSIBLE INVESTIGATORS (List only those under DFCI IRB, *i.e.*, from institutions listed in Section 6 below)

**Overall PI:**	Jeffrey Meyerhardt, MD, MPH	**Phone:**	617-632-6855	**Institution(s):**	DFCI
**Site Responsible PI:**	Andrew Zhu, MD	**Phone:**	617-724-0786	**Institution(s):**	MGH
	Michael Goldstein, MD		617-667-4852		BIDMC

Co-Investigators name (institution): Matthew Kulke, MD/DFCI, Ramesh Shivdasani, MD/DFCI, Robert Mayer, MD/DFCI, Lawrence Blaszkowsky, MD/MGH, Eunice Kwak, MD/MGH, Jeffrey Clark, MD/MGH, Michael Goldstein, MD/BIDMC, David Ryan, MD/MGH, Charles Fuchs, MD/DFCI,
Peter Enzinger, MD/DFCI, Jill Allen, MD/MGH, Pankaj Bhargava, MD/DFCI, Thomas Abrams, MD/DFCI, Jennifer Ang Chan, MD/DFCI, Brian Wolpin, MD/DFCI, Kimmie Ng, MD/DFCI, Deborah Schrag, MD/DFCI, Raymond Wadlow, MD/MGH, Wolfram Goessling, MD/DFCI, Alexi Wright,
MD/DFCI; Robin Sommers, NP/DFCI, Mary Rapp, NP/DFCI, Nina Grenon, NP/DFCI,Theresa McDonnell, NP/MGH, Emily Olson, NP/MGH, Michelle Knowles, NP/MGH, Rhea Photopoulos,NP/MGH

Additional Contacts (Please list group mailbox email addresses; if listing an individual, please list no more than one person from the non-lead institutions listed in section 6 below):usan Sheehan, RN/MGH, Kathryn Componeschi/BIDMC

3. DRUG / DEVICE INFORMATION N/A:

Drugs, Biologics, Devices (name & IND/IDE#): Zactima (ZD6474 IND#75234)

IND/IDE held by: Jeffrey Meyerhardt, MD, MPH (Check if IND/IDE exempt: ☐)

Investigational Drug Brochure (IDB) Version No./ Date: Cetuximab IB Version 14 (8/24/2009)
Vandetanib (ZD6474) IB Edition 13 (9/22/2011)
(Check if already on file with OHRS:☐)

4. PROTOCOL COORDINATION, FUNDING, PHASE, MODE, TYPE ETC.

Protocol Coordinated By:
DF/HCC Investigator Jeffrey Meyerhardt, MD, MPH

Cancer Related: Yes If yes:
Primary Disease Program:
Gastrointestinal Malignancies
or
Primary Discipline Based Program:
Chemoprevention

Funding/Support (check all that apply):
☒ Industry: AstraZeneca
☐ Federal Organization:
Grant #:
☐ Internal Funding:
☐ Non-Federal:
☐ Other:

Phase: Phase 1

Multi-Center (i.e., non-DF/HCC site participation):
No

Protocol Type: Therapeutic
If Ancillary, provide parent protocol #:

Protocol Involves (check all that apply as listed in the protocol document, even if not part of the research but is mandated by the protocol document):

☐ Chemotherapy	☐ Hormone Therapy	☒ Medical Record Review
☐ Immunotherapy	☐ Vaccine	☐ Questionnaires/Surveys/Interviews
☐ Surgery	☒ Data Repository	☒ Radiological Exams
☐ Bone Marrow/Stem Cell Transplant	☐ Exercise/Physical Therapy	☐ Required Biopsy Study
☐ Cell Based Therapy	☐ Genetic Studies	☐ Human Embryonic Stem Cell
☐ Gene Transfer (use of recombinant DNA)	☐ Human Material Banking	☒ Other: patient diaries
☐ Radiation Therapy	☒ Human Material Collection	

5. SUBJECT POPULATION (also applies to medical record review and specimen collection studies)

Total Study-Wide Enrollment Goal: 34 **Greater than 25% of the overall study accrual will be at DF/HCC:** ☒ Yes ☐ No

Total DF/HCC Estimated Enrollment Goal: 34 **Adult Age Range:** 18+ **Pediatric Age Range:** NA

Will all subjects be recruited from pediatric clinics? ☐ Yes ☒ No

Protocol Front Sheet

If enrolling both adults and pediatric subjects, anticipated percent of pediatric subjects: NA

Retrospective Medical Record Reviews (Please provide date range): NA

6. INSTITUTIONAL PARTICIPANTS UNDER DFCI IRB

Dana-Farber/Harvard Cancer Center: (check all that apply)

- ☒ Beth Israel Deaconess Medical Center
- ☒ Brigham and Women's Hospital
- ☐ Children's Hospital Boston
- ☒ Dana-Farber Cancer Institute
- ☒ Dana-Farber Cancer Institute at Faulkner Hospital
- ☐ Dana-Farber Cancer Institute at Londonderry Hospital
- ☐ Dana-Farber Cancer Institute at Milford Hospital
- ☐ Dana-Farber Cancer Institute at South Shore
- ☒ Massachusetts General Hospital
- ☐ Massachusetts General Hospital/North Shore Cancer Center
- ☐ Massachusetts General Hospital Radiation Oncology at Emerson Hospital

DF/PCC Network Affiliates: (check all that apply)

- ☐ Cape Cod Healthcare (Hyannis, MA; Mashpee, MA)
- ☐ Lowell General Hospital (Lowell, MA)
- ☐ New Hampshire Oncology-Hematology-P.A. (Concord, NH; Hooksett, NH; Laconia, NH)
- ☐ Newton-Wellesley Hospital (Newton, MA)

7. DF/HCC INITIATED STUDIES ONLY - INSTITUTIONAL PARTICIPANTS UNDER OTHER IRB (N/A:)

DF/HCC Multi-Center Protocols: (list institution/location)

DF/PCC Network Affiliates: (list institution/location)

8. OTHER INVOLVED SITES NOT UNDER DFCI IRB (N/A:)

Please list other sites that will be involved in portions of the study, for example, ocular screening and monitoring that will be conducted at Joslin Diabetes Center: (list institution/location)

Protocol Number: 06-177

Approval Date: 08/29/06 (IRB meeting date when protocol/consent approved or conditionally approved)

Activation Date: 02/12/07 (Date when protocol open to patient entry)

Approval signatures are on file in the Office for Human Research Studies, tel. 617-632-3029.

Date Posted	Revised Sections	IRB Approval Date
02/16/07	Delay Activation: MGH ready	N/A
03/06/07	Delay Activation: BIDMC ready	N/A
08/03/07	Consent Form replaced due to Continuing Review # 1	07/19/07
08/28/07	Consent Form, Protocol and Front Sheet replaced due to Amendment #2	08/10/07
09/18/07	Temporary closure to enrollment until it is determined if a dose limiting toxicity has occurred by the end of patients first cycle (Effective 09/11/07). Reference Amendment #3	09/18/07
09/25/07	Re-open to accrual due to Amendment #5	09/25/07
11/20/07	Consent Form, Protocol and Front Sheet replaced due to Amendment #4	11/06/07
12/12/07	Alert Page replaced due to Amendment #6	11/28/07
01/16/08	Protocol and Front Sheet replaced due to Amendment #7	01/13/08
02/22/08	Alert Page and Consent Form replaced due to Amendment # 8	01/09/08
03/12/08	Alert page replaced due to Amendment #10	03/12/08
06/25/08	Study renewal due to Continuing Review #2	06/12/08
07/17/08	Front Sheet and Protocol replaced due to Amendment #11	06/05/08
07/17/08	Consent Form, Protocol and Front Sheet replaced due to Amendment #12	07/09/08
07/17/08	Front Sheet replaced due to Amendment #9	07/15/08
02/13/09	Alert Page and Front Sheet replace due to Amendment #13	02/02/09
03/17/09	Temporary closure to enrollment until it is determined if a dose limiting toxicity has occurred by the end of patients first cycle (Effective 03/16/09; reference Amendment #14)	03/17/09
04/08/09	Re-open to enrollment; Alert page replaced due to Amendment #15	04/08/09
05/22/09	Consent Form and Alert page replaced due to Amendment #16, and Adverse Event # 4	05/18/09
06/08/09	Consent Form (footer) replaced due to Continuing Review # 3	05/14/09
08/11/09	Consent Form, Protocol and Front Sheet replaced due to Amendment #17	07/21/09
08/12/09	Alert Page replaced due to Amendment #18	08/11/09

08/24/09	Front Sheet replaced due to Amendment #19	08/21/09
11/03/09	Protocol, Consent Form and Front Sheet replaced due to Amendment #20	10/20/09
03/08/10	Front Sheet replaced due to Amendment #22	01/29/10
05/13/10	Study renewal due to Continuing Review #4	04/22/10
05/20/10	Permanent closure to new accrual: due to target enrollment goal met (effct. 05/18/10; Amendment #23)	05/20/10
08/18/10	Front Sheet replaced due to Amendment #25	08/10/10
10/21/10	BIDMC site ON HOLD due BIDMC Strategic Hold OE #16	N/A
04/11/11	Protocol, Consent Form (CR 4 approval date), Alert Page and Front Sheet replaced due to Amendment #21	01/07/10
04/19/11	Study renewal/ Consent Form footer and Front Sheet replaced due to Continuing Review #5	04/13/11
12/27/11	Front Sheet replaced due to Amendment #26	12/16/11
04/10/12	Study renewal/ Consent Form footer replaced due to Continuing Review #6	04/09/12

Phase I Study of ZD6474, Cetuximab, and Irinotecan in Patients with Metastatic Colorectal Cancer

Coordinating Center: Dana-Farber/Harvard Cancer Care

Participating: Institutions

Dana-Farber Cancer Institute/Brigham & Women's Hospital
Overall Principal Investigator: Jeffrey A. Meyerhardt, MD, MPH

Massachusetts General Hospital
Site Principal Investigator: Andrew X. Zhu, MD

Beth Israel Deaconess Medical Center
Site Principal Investigator: Michael Goldstein, MD

Principal Investigator:

Jeffrey A. Meyerhardt, MD, MPH
Dana-Farber Cancer Institute
44 Binney Street
Boston, MA 02115
617-632-6855
jmeyerhardt@partners.org

Co-Investigator:

Andrew X. Zhu, MD, PhD
Medical Oncology Unit
Massachusetts General Hospital
55 Fruit Street
Boston, MA 02114
617-724-0786
azhu@partners.org

Co-Investigator:

Michael Goldstein, MD
Beth Israel Deaconess Medical Center
330 Brookline Ave
Boston, MA 02115
617-667-9235
sjain@bidmc.harvard.edu

Correlative Sciences: (biomarkers/CEC)

Rakesh K. Jain, Ph.D.
Massachusetts General Hospital
100 Blossom Street
617-726- 8143
jain@steele.mgh.harvard.edu

**Correlative Sciences: (tumor blocks)**	Shuji Ogino<br>Dana Farber Cancer Institute<br>44 Binney Street<br>Boston, MA 02115<br>617-632-3978<br>sogino@partners.org
**Responsible Research Nurses:**	Eileen Regan, RN<br>Dana-Farber Cancer Institute<br>44 Binney Street<br>Boston, MA 02115<br>617-632-3898<br>eileen_regan@dfci.harvard.edu
**Responsible Data Manager:**	Brittany Allen<br>Dana-Farber Cancer Institute<br>44 Binney Street<br>Boston, MA 02115<br>617-632-6746<br>BAllen2@partners.org

PROTOCOL SYNOPSIS

TITLE	Phase I Study of ZD6474, Cetuximab, and Irinotecan in Patients with Metastatic Colorectal Cancer
INVESTIGATORS / TRIAL LOCATION	Dana-Farber Cancer Institute/Brigham & Women's Hospital<br>Overall principal Investigator: Jeffrey A. Meyerhardt, MD, MPH<br><br>Massachusetts General Hospital<br>Site Principal Investigator: Andrew X. Zhu, MD, PhD<br><br>Beth Israel Deaconess Medical Center<br>Site Principal Investigator: Michael Goldstein, MD
STUDY OBJECTIVES	PRIMARY<br>The primary objective of this study will be to determine the tolerability and maximum tolerated dose (MTD) of combining ZD6474, cetuximab and irinotecan in patients with metastatic colorectal cancer refractory to prior cytotoxic chemotherapy.<br><br>SECONDARY<br><br>Determine response rate, progression-free survival and overall survival of adding ZD6474 to cetuximab and irinotecan in patients with metastatic colorectal cancer previously treated.<br><br>EXPLORATORY<br><br>We will explore whether molecular alterations within tumor blocks in VEGF, CD31 (for microvessel density), EGFR, p21, p53, p-AKT1, MSI, K-ras, and BRAF with responsiveness to therapy to seek for any tissue biomarker to predict responsiveness or resistance to the combination under study. We will explore changes in serum VEGF and other angiogenesis cytokine markers before and after initiation of ZD6474. See Appendix C.
STUDY DESIGN	Phase I
STUDY POPULATION	INCLUSION CRITERIA<br>Patients must meet all of the following criteria in order to be eligible for entry into the trial:<br>(1) Eligible patients must have histologically or cytologically documented locally advanced or metastatic colorectal cancer<br>(2) 1-2 prior therapies for metastatic colorectal cancer. Prior adjuvant therapy not included in the number of priors unless recurrence within 12 months of last dose of therapy and then the adjuvant therapy will be counted as 1 prior.<br>a. Patients must have measurable disease according to

the RECIST criteria.
 b. Tumor is K-ras wildtype by method of choice at respective institution (testing codons 12 and 13)

(3) Age > 18 years
(4) ECOG Performance Score of 0-2.
(5) Patients should have completed any major surgery 4 weeks from registration. Patients must have completed any minor surgery 2 weeks from registration. Patients must have fully recovered from the procedure and have completely healed surgical incisions before starting therapy. (Insertion of a vascular access device is not considered major or minor surgery.)
(6) Adequate bone marrow as evidenced by:
 Absolute neutrophil count ≥ 1,200/mL
 Platelet count ≥ 100,000/mL
(7) Adequate renal function as evidenced by serum creatinine ≤1.5 x ULRR or creatinine clearance ≥ 50 mL/minute (calculated by Cockcroft-Gault formula)
(8) Adequate hepatic function as evidenced by:
 Serum total bilirubin <= 1.5 mg/dL
 Alkaline phosphatase < 3X the ULN for the reference lab (< 5X the ULN for patients with known hepatic metastases)
 SGOT/SGPT < 3X the ULN for the reference lab (< 5X the ULN for patients with known hepatic metastases)
(9) Normal range of serum calcium (ionized or adjusted for albumin) and magnesium (supplementation permitted)
(10) Patients or their legal representatives must be able to read, understand and provide informed consent to participate in the trial.
(11) Patients of childbearing potential and their partners must agree to use an effective form of contraception during the study and for 90 days following the last dose of study medication (an effective form of contraception is an oral contraceptive or a double barrier method)
(12) For women who have not had a hysterectomy and under 60 years of age, a negative urine or serum pregnancy test . Male patients must be surgically sterile or using an acceptable method of contraception during their participation in this study.

EXCLUSION CRITERIA
Patients with any of the following criteria are ineligible for entry:
(1) History of prior malignancy within the past 3 years except for curatively treated basal cell carcinoma of the skin, cervical intra-epithelial neoplasia, or treated

localized prostate cancer with a current PSA of < 1.0 mg/dL on 2 successive evaluations, at least 3 months apart, with the most recent evaluation no more than 4 weeks prior to entry.

(2) Prior therapy with gefitinib, erlotinib, cetuximab, ABX-EGF or other specific EGFR inhibitor. Note that prior therapy with either bevacizumab and/or irinotecan is NOT an exclusion criteria

(3) Patients with known hypersensitivity to any of the components of cetuximab or ZD6474 or irinotecan

(4) Patients who are pregnant or lactating

(5) Any other medical condition, including mental illness or substance abuse, deemed by the clinician to be likely to interfere with a patient's ability to sign informed consent, cooperate and participate in the study, or interfere with the interpretation of the results.

(6) Potassium <4.0 mEq/L despite supplementation;

(7) Serum calcium (ionized or adjusted for albumin,) or magnesium out of normal range despite supplementation.

(8) Evidence of severe or uncontrolled systemic disease or any concurrent condition which in the Investigator's opinion makes it undesirable for the patient to participate in the trial or which would jeopardize compliance with the protocol.

(9) Clinically significant cardiac event such as myocardial infarction; New York Heart Association (NYHA) classification of heart disease ≥ 2 that, in the opinion of the Investigator, increases the risk of ventricular arrhythmia (see Appendix B) within 3 months before entry; or presence of cardiac disease

(10) History of arrhythmia (multifocal premature ventricular contractions (PVCs), bigeminy, trigeminy, ventricular tachycardia, or uncontrolled atrial fibrillation) which is symptomatic or requires treatment (CTCAE grade 3) or asymptomatic sustained ventricular tachycardia. Atrial fibrillation, controlled on medication is not excluded.

(11) Previous history of QTc prolongation as a result from other medication that required discontinuation of that medication.

(12) Congenital long QT syndrome,or 1st degree relative with unexplained sudden death under 40 years of age.

(13) Presence of left bundle branch block (LBBB.)

(14) QTc with Bazett's correction that is unmeasurable, or ≥480 msec on screening ECG. If a patient has QTc ≥480 msec on screening ECG, the screen ECG may be repeated twice. The average QTc from the three

	screening ECGs must be <480 msec in order for the patient to be eligible for the study). QTC>460 msec for patients taking medications known to induce Torsades de Pointes (see Appendix A, Drug List 1; section 5.2)<br>(15) Any concomitant medication known to be a potent inducer of CYP3A4 function; for a list of medications which may affect CYP3A4 activity, please visit: http://www.medicine.iupui.edu/clinpharm/ddis/ClinicalTable.asp and http://www.pharmacytimes.com/issue/pharmacy/2008/2008-09/2008-09-8687.<br>(16) Hypertension not controlled by medical therapy (systolic blood pressure greater than 160 mm Hg or diastolic blood pressure greater than 100 mm Hg)<br>(17) Patients lacking physical integrity of the upper gastrointestinal tract or who have malabsorption syndrome.<br>(18) Currently active diarrhea that may affect patient's ability to absorb ZD6474 or tolerate potential diarrhea from study drugs.<br>(19) Receipt of any investigational agents within 30 days prior to commencing study treatment<br>(20) Last dose of prior chemotherapy or radiation therapy discontinued less than 4 weeks before the start of study therapy<br>(21) Incompletely healed surgical incisions, at the discretion of the investigator.<br>(22) Any unresolved toxicity greater than CTC grade 1 from previous anti-cancer therapy<br>(23) EGFR by immunohistochemistry will NOT be an exclusion
Total expected number of patients	3-34
Expected number of centers	3
Dose LEVELS	Phase I trial with sequential dose escalation of ZD7474 and irinotecan. We will initially test the tolerability of lowest dose of ZD6474 with standard dosing of cetuximab in dose level 1. If this combination is tolerable, then we will add standard dose every other week irinotecan and test for tolerability. We will then dose escalate ZD6474 with standard weekly cetuximab and every other week irinotecan.<br>Dose levels — ZD6474 — Irinotecan<br>1 — 100 mg — --<br>2 — 100 mg — 180 mg/m2<br>3 — 200 mg — 180 mg/m2<br>4 — 300 mg — 180 mg/m2

Dose LIMITING TOXICITIES	DLTs (defined as within the first 28 days of treatment in dose level 1 and first 35 days in dose levels 2-4) will include: grade IV hematological toxicity > 7 days; fever and neutropenia; grade III diarrhea leading to hospitalization or lasting > 48 hours despite aggressive anti-diarrheal medication; grade IV diarrhea despite aggressive anti-diarrheal medication; grade IV vomiting despite optimal antiemetics; or grade III or higher nonhematological toxicity (excluding nausea, vomiting, diarrhea, or alopecia) lasting > 1 week; grade 4 skin toxicity; grade 3 or greater cardiac toxicities; or death.

STUDY SCHEMA (REVISED)

Patients sign inform consent

Enter current dose level

Dose levels	ZD6474	Irinotecan	Cetuximab †
1	100 mg	--	250 mg/m2
2	100 mg	180 mg/m2	250 mg/m2
3	200 mg	180 mg/m2	250 mg/m2
4	300 mg	180 mg/m2	250 mg/m2

† - after 400 mg/m2 loading dose

3 patients treated –
If no DLTs, next dose level
If 1 DLT, enter 3 more patients at that level
- If < 33% have DLT (1/6) then next dose level
- If 2 or more have DLT, no dose level advancement –
  - ❖ If at dose level 1, study closes.
  - ❖ Else, MTD is current dose level -1

Treat 10 additional patients at MTD

NOTE: Patients at all dose levels initiate ZD6474 and Cetuximab on day 1. Patients on dose levels 2-4 (with irinotecan) with start first 2 weeks with ZD6474 and Cetuximab only then start Irinotecan on day 15.

Table of Contents

Protocol Synopsis **3**
Schema **8**

1. BACKGROUND **11**
   - **1.1 COLORECTAL CANCER 11**
   - **1.2 TARGETED THERAPY 11**
   - **1.3 EPIDERMAL GROWTH FACTOR RECEPTOR 11**
   - **1.4 VASCULAR ENDOTHELIAL GROWTH FACTOR 12**
   - **1.5 CURRENT TREATMENT SCHEMAS 12**
   - **1.6 COMBINING TARGETED AGENTS 12**
   - **1.7 ZD6474 13**
   - **1.8 ADVERSE EVENTS** in **ZD6474 (ZD6474) LUNG CANCER STUDIES 13**
   - **1.9 RATIONALE 15**

2. OBJECTIVES **16**
   - **2.1 PRIMARY OBJECTIVE 16**
   - **2.2 SECONDARY OBJECTIVES 16**
   - **2.3 EXPLORAORY OBJECTIVES 16**

3. STUDY SPECIFICATIONS **16**
   - **3.1 STUDY DESIGN 16**
   - **3.2 DOSE LIMITING TOXICITIES 18**
   - **3.3 EXPECTED NUMBER OF PATIENTS 18**
   - **3.4 METHOD OF TREATMENT ALLOCATION 18**
   - **3.5 DURATION OF THERAPY 18**

4. STUDY POPULATION **19**
   - **4.1 INCLUSION CRITERIA 19**
   - **4.2 EXCLUSION CRITERIA 20**
   - **4.3 RESTRICTION 21**

5. STUDY PROCEDURES **21**
   - **5.1 TREATMENT REGIMEN 21**
   - **5.2 SUPPORTIVE THERAPIES 22**

6. DRUG FORMULATIONS AND SAFETY **24**
   - **6.1 ZD6474 (ZACTIMA) 24**
   - **6.2 IRINOTECAN 26**
   - **6.3 CETUXIMAB 28**

7. DOSE AND SCHEDULE MODIFICATIONS **31**
   - **7.1 ZD6474 DOSE MODIFICATIONS 32**
   - **7.2 CETUXIMAB DOSE MODIFICATIONS 36**
   - **7.3 IRINOTECAN DOSE REDUCTIONS 38**

8. ADVERSE EVENTS **40**
8.1 MONITORING OF ADVERSE EVENTS 40
8.2 EVENTS DEFINITIONS 40
8.3 RECORDING AND REPORTING 41

9. STUDY CALENDAR **43**
9.1 PRE-REGISTRATION WORK-UP 43
9.2 EVALUATION DURING TREATMENT 44
9.3 POST-TREATMENT FOLLOW-UP 44

10. REQUIRED DATA **45**

11. PATIENT WITHDRAWAL AND STUDY TERMINATION **46**

12. MEASUREMENT OF EFFECT **46**
12.1 ELIGIBILITY 47
12.2 METHODS AND MEASUREMNT 47
12.3 BASELINE DOCUMENTATION OF "TARGET" AND "NON-TARGET" LESIONS 48
12.4 RESPONSE CRITERIA 48
12.5 EVALUATION OF BEST OVERALL RESPONSE 49
12.6 CONFIRMATION 49
12.7 DURATION OF OVERALL RESPONSE 50
12.8 REPORTING OF RESULTS 50

13. STATISTICAL CONSIDERATIONS STUDY **50**

14. ETHICAL AND REGULATORY CONSIDERATIONS **51**
14.1 ETHICAL PRINCIPLES 51
14.2 INFORMED CONSENT 51
14.3 INSTITUTIONAL REVIEW BOARD (IRB) APPROVAL 51
14.4 ADDITIONAL RESPONSIBILITIES OF THE INVESTIGATOR 52
14.5 CONFIDENTIALITY 52
14.6 PROTOCOL AMENDEMENT 53
14.7` AUDITS 53
14.8 PATIENT DATA PROTECTION 53

16. REFERENCES **54**

APPENDIX A. MEDICATIONS KNOWN TO PROLONG THE INTERVAL AND/OR INDUCE TORSADES DE POINTES (TDP) **56**
APPENDIX B. NEW YORK HEART ASSOCIATION (NYHA) CARDIAC CLASSIFICATION **60**
APPENDIX C. CORRELATIVE SCIENCE **61**

1 BACKGROUND

1.1 COLORECTAL CANCER

Colorectal cancer is the third most common malignancy and second most frequent cause of cancer-related death in the United States, with 145,270 new cases and 56,290 deaths anticipated in 2005. Worldwide, colorectal cancer is the fourth most commonly diagnosed cancer, with an estimated 498,000 new cases and 255,000 deaths each year.[1] Up to 20% of patients present with metastatic disease at the time of diagnosis and 40% will eventually develop advanced disease. Metastatic colorectal cancer is not curable. Chemotherapy is palliative, improving symptoms and overall survival.[2-5]

In the past 5 years, a host of newer agents have been tested and introduced in the treatment of metastatic colorectal cancer, including irinotecan, oxaliplatin, bevacizumab and cetuximab. Patients now have a variety of options of first, second and third line therapy. However, ultimately, none of these combinations are curative and innovative approaches are necessary.

1.2 TARGETED THERAPY

Laboratory studies have identified molecular sites in tumor tissue that may serve as specific targets for treatment. The goal of such a therapeutic strategy is the interruption of cellular pathways essential for tumor growth, survival and metastasis, while potentially reducing toxicities associated with less specific cytotoxic chemotherapies. At present, two classes of such targeted compounds have been introduced in the management of patients with advanced colorectal cancer: epidermal growth factor receptor antagonists and angiogenesis inhibitors.[6]

1.3 EPIDERMAL GROWTH FACTOR RECEPTOR

The epidermal growth factor receptor (EGFR) is a transmembrane glycoprotein that is involved in signaling pathways affecting cellular growth, differentiation, proliferation and programmed cell death.[7] EGFR is present on the surface of normal epithelium and is overexpressed in certain tumors. Such overexpression has been associated with a poorer prognosis in colorectal cancer.[8] EGFR inhibition can be achieved by antibodies directed against the extracellular domain or the soluble ligands of the receptor, inhibitors to the required dimerization of the receptor or small molecules which prevent phosphorylation of the receptor by its intracellular tyrosine kinase.

Preclinical studies have not only demonstrated therapeutic synergy between cetuximab and chemotherapy, but that such synergy can occur in tumor cells already resistant to irinotecan, suggesting that the inhibitor may overcome cellular resistance to irinotecan.[7] As such, Saltz and colleagues treated 121 patients with advanced colorectal cancer whose tumor had been shown unresponsive to irinotecan therapy with the combination of cetuximab and irinotecan; 19 percent of patients experienced significant tumor shrinkage.[9] To determine whether this antitumor effect was due to

the synergy between the two drugs or to the independent activity of cetuximab, 60 similar patients were treated with only the antibody; 10 percent of these individuals had significant tumor regression.[10] These experiences were confirmed and extended by Cunningham and colleagues who randomized 329 patients with advanced colorectal cancer refractory to irinotecan to receive either cetuximab with irinotecan or cetuximab alone.[11] This larger clinical trial resulted in an almost identical 23 percent rate of disease regression in patients given the combination and 11 percent in those who received single-agent cetuximab.

1.4 VASCULAR ENDOTHELIAL GROWTH FACTOR

The appreciation that tumors induce blood vessel formation to allow extension beyond a few millimeters in size stimulated efforts at inhibiting such angiogenesis as a means of controlling the growth and spread of cancer cells.[12] The most successful of these efforts to date has focused on neutralizing the vascular endothelial growth factor (VEGF), which is a soluble protein instrumental in the angiogenesis process.[13] Bevacizumab (Avastin™) is a humanized antibody directed against the vascular endothelial growth factor that has been examined in combination with chemotherapy in several clinical trials in patients with advanced colorectal cancer.[14-16] A small randomized phase II trial in patients who had received no prior treatment for their metastatic disease demonstrated that bevacizumab improved the likelihood of tumor response compared to fluorouracil and leucovorin alone.[15] This effort led to two concurrent randomized phase III trials. Hurwitz and colleagues allocated 815 patients to receive IFL with either bevacizumab or placebo.[16] The addition of bevacizumab led to an impressive statistically significant increase in response rate and a 4.7 months prolongation of median overall survival (15.6 months to 20.3 months).

1.5 CURRENT TREATMENT SCHEMAS

Patients with metastatic colorectal cancer who have a good performance status are currently offered combined modality therapy incorporating cytotoxic chemotherapies with therapies targeted against EGFR or VEGF. In the first line, combinations of bevacizumab with either 5-FU, leucovorin and irinotecan (IFL or FOLFIRI) or 5-FU, leucovorin and oxaliplatin (FOLFOX) are common.[6] Second and third line therapy depend on the choice of first line therapy, but usually incorporate cetuximab at some point and the cytotoxic therapy not utilized yet (irinotecan and cetuximab).

1.6 COMBINING TARGETED AGENTS

These has been recent interest in combining targeted agents to maximize directed inhibition of tumor growth. Saltz and colleagues recently presented data combining cetuximab, bevacizumab and irinotecan in patients who had prior chemotherapy for metastatic colorectal cancer but were not previously exposed to cetuximab or bevacizumab (ASCO 2005). They demonstrated an impressive response rate of 37% with a progression free survival of 8 months, considering most patients had at least 2 prior regimens of therapy.

Preclinical experiments have supported combining EGFR monoclonal antibodies with an oral inhibitor of EGFR TK. Matar et al demonstrated that combined therapy of gefitinib and cetuximab resulted in synergistic effects on proliferation and induction of apoptosis in a variety of cell lines (colon, vulvar, prostate and breast) and an EGFR-dependent human tumor xenograft model.[17] Huang and colleagues demonstrated similar findings with the combination using a head and neck cell line.[18] Phosphorylation inhibition of downstream effector molecules (MAPK and AKT) were enhanced by combining gefitinib and cetuximab.

1.7 ZD6474

Newer generations of directed therapy appear to inhibit multiple targets in the cancer mechanism. ZD6474 (Zactima) is receptor tyrosine kinase (RTK) inhibitor that, in isolated enzyme assays, potently inhibits vascular endothelial growth factor receptor-2 (VEGFR-2) tyrosine kinase activity (IC50 = 40 nM), and shows additional inhibitory activity at sub-micromolar concentrations against Rearranged during Transfection (RET) receptor tyrosine kinase (inhibitory concentration [IC50] = 100 nM), Flt-4 (VEGF receptor-3: IC50 = 110 nM) and EGFR (IC50 = 500 nM) tyrosine kinases.[19,20] It is expected that this molecule may be beneficial in a broad range of human malignancies, and perhaps other diseases, that are dependent upon VEGF-mediated angiogenesis or EGF and RET mediated tumor growth. ZD6474 has shown excellent reversible inhibition of tumor cell growth in a broad range of pre-clinical models, including lung cancer and thyroid cancer xenografts. Regression of some established tumors in animals were observed following oral administration. Pre-clinical toxicology shows the agent to be well tolerated after 6 months of daily administration.

Two Phase I studies were conducted in the West and in Japan, which demonstrated a maximum tolerated dose of 300 mg, with common adverse events (AEs) being diarrhea, rash, and asymptomatic QTc prolongation. In phase II development, studies have been conducted in NSCLC, breast cancer, and multiple myeloma with ZD6474 monotherapy at 300mg. In addition, a randomized double-blind study was conducted to compare ZD6474 at 100 mg or 300 mg in combination with Docetaxel to Docetaxel plus placebo. In Study ZD6474IL/0003 (the largest monotherapy study conducted thus far) and Study ZD6474IL/0006 (chemo-combination with Docetaxel), both studies demonstrated that ZD6474 prolonged progression-free survival (PFS) in patients with NSCLC. (Natale R, ASCO 2005; Heymach J, ASCO 2005.)

1.8 ADVERSE EVENTS in ZD6474 (ZD6474) Lung Cancer Studies

The most common AEs associated with ZD6474 in the phase I and other monotherapy studies included rash, diarrhea and asymptomatic QTc prolongation. In Study ZD6474IL/0003 (the largest monotherapy ZD6474 study conducted as of this date,) subjects with locally advanced or metastatic (IIIB/IV) non-small cell lung cancer were enrolled after failure of first-line and/or second-line platinum-based chemotherapy. In Part A, subjects were randomized to 1 of 2 double-blind treatment arms; 300 mg ZD6474, or 250 mg gefitinib. The most frequent AEs for ZD6474 (Part

A) were diarrhea (55.4%), fatigue (36.1%), rash (27.7%), and nausea (24.1%). Approximately 10% of subjects who received ZD6474 had an adverse event of hypertension (HTN). The majority were Common Toxicity Criteria (CTC) grade 1 or 2. Three subjects developed CTC grade 3 HTN, and none were CTC grade 4. There were no serious adverse events (SAEs) of HTN. The median increase in systolic blood pressure for subjects who received ZD6474 was 10 mmHg; the median increase in diastolic blood pressure was 6 mmHg.

An increased incidence of SAEs was noted in subjects who received ZD6474 compared to those who received gefitinib (44.6% vs. 35.3%.) Cardiac disorders (6.0% vs. 1.2%), gastrointestinal disorders (6% vs. 2.4% mainly diarrhea,) and respiratory disorders (13.3% vs. 8.2%) did occur more frequently in subjects receiving ZD6474. The cardiac events included a variety of terms without any apparent pattern. Respiratory events were primarily those that would be anticipated in subjects with advanced lung cancer. Three subjects receiving ZD6474 developed pulmonary embolism and 3 subjects developed interstitial lung disease, but cases were confounded by such factors as smoking, reduced mobility, infection, lung cancer progression and previous chemotherapy or radiation therapy. One subject in each arm developed a serious skin disorder. One subject who received ZD6474 developed a hematologic event, as did 2 subjects who received gefitinib. No subjects who received ZD6474 developed serious hepatotoxicity.

At the time of data cut-off, 49.4% of subjects in the ZD6474 arm and 42.4% of subjects in the gefitinib arm had died. The primary cause of death was disease progression. Other causes of death for the ZD6474 arm included acute respiratory distress syndrome (ARDS) (n=1), choking (n=1), interstitial lung disease (n=1), large intestine perforation (n=1), and sepsis (n=1). Thirteen adverse events on study were followed by an outcome of death, 7 in the ZD6474 arm (ARDS, pneumonia, pneumonitis, dyspnea, interstitial lung disease, respiratory failure and carcinomatous meningitis).

In Study 6474IL/0006, patients with advanced or metastatic NSCLC were enrolled after failure of prior platinum-based chemotherapy. Patients were randomized to treatment with a standard dose of docetaxel and either placebo or 100 mg of ZD6474 or 300 mg of ZD6474.

The most frequent AEs observed in this study were similar to those observed in prior trials for ZD6474 or reported for Docetaxel in the literature. The most common AEs and their frequencies as reported in the 300 mg ZD6474, 100 mg ZD6474 and placebo arms, respectively, were diarrhea (50.0%, 38.1%, 24.4%), fatigue (45.5%, 40.5%, 26.8%), neutropenia (31.8%, 26.2%, 19.5%) and nausea (29.5%, 26.2%, 19.5%). Rash was observed in 15.9%, 16.7% and 9.8% of patients in the three arms.

Gastrointestinal events (77.3%, 59.5%, 41.5%), and skin events (72.7%, 64.3%, 41.5%) were more common in the ZD6474-containing arms. For all arms, the majority of these events were common toxicity criteria (CTC) grade 1 and 2; however CTC grade 3/4 events were more prominent in the ZD6474 300 mg arm. Cardiac

disorders occurred more frequently in ZD6474-containing arms (15.9%, 14.3%, 2.4%.). The majority were CTC grade 1/2 and included a variety of terms, none ventricular. The frequency of respiratory events (50.0%, 57.1%, 46.3%) was similar in all arms. Neutropenia (31.8%, 26.2%, 19.5%) and related terms were more common in ZD6474-containing arms, but this did not result in an increase in infection. There was little difference in frequency of other hematologic events.

Approximately 10% of patients receiving ZD6474 developed an AE of hypertension. The majority of events were CTC grade 1 or 2, and no events were CTC grade 4. There were no serious adverse events (SAEs) of hypertension. The maximum median rise in both systolic and diastolic blood pressure was approximately 8 mm Hg in patients who received ZD6474 300 mg, and approximately 4 mm Hg in patients who received ZD6474 100 mg.

There were 12 subjects with confirmed QTc prolongation in Study ZD6474IL/0003. Of these, 6 occurred in the first 28 days, and 2 in the following 28 days. The remaining 4 occurred sporadically, with the longest time to occurrence 323 days. There were 3 events of CTC grade 1 reversible dizziness in subjects with a confirmed QTc prolongation occurring within the first 4 weeks. Subjects with dizziness had other events that might have caused dizziness and the events were not well correlated in time with the actual QTc prolongation. There were no other potentially relevant adverse events in subjects with confirmed QTc prolongation within the first 4 weeks, and no relevant adverse events in subjects whose first confirmed QTc prolongation occurred more than 4 weeks after randomization.

In Study ZD6474IL/0006, 7 patients had confirmed QTc prolongation. Five (16%) occurred on the ZD6474 300 mg/Docetaxel arm, 4 of which occurred in the first 28 days, and 1 at day 70. Two (12%) occurred on the ZD6474 100 mg /Docetaxel arm, at days 22 and 43. No patients with confirmed QTc prolongation in study 6474IL/0006 experienced a potentially relevant AE. One patient in the run-in phase of trial 6 had received ZD6474 300 mg plus Docetaxel, was hospitalized with post-obstructive pneumonia as well as hypokalemia resulting from Prednisone and Fluorinef given to treat adrenal insufficiency resulting from adrenal metastases. He was noted to have a QTc interval of 626 millisecond (msec.) During this hospitalization he developed a 12 beat run of ventricular tachycardia, which was asymptomatic and resolved without treatment.

1.9 RATIONALE

Patients with metastatic colorectal cancer are often treated with cetuximab +/- irinotecan in the 2nd or 3rd line setting. While some patients will have reasonably prolonged disease control, innovative strategies are still needed in previously-treated patients with metastatic colorectal cancer. We seek to determine the tolerability of combining a multi-targeted agent (ZD6474) with cetuximab and irinotecan in such a patient population and get preliminary data on the activity of such combinations.

2 OBJECTIVES

2.1 PRIMARY

The primary objective of this study will be to determine the tolerability and maximum tolerated dose (MTD) of combining ZD6474, cetuximab and irinotecan in patients with metastatic colorectal cancer refractory to prior cytotoxic chemotherapy.

2.2 SECONDARY

Determine response rate, progression-free survival and overall survival of adding ZD6474 to cetuximab and irinotecan in patients with metastatic colorectal cancer previously treated.

2.3 EXPLORATORY

We will explore whether molecular alterations within tumor blocks in VEGF, CD31 (for microvessel density), EGFR, p21, p53, p-AKT1, MSI, K-ras, and BRAF with responsiveness to therapy to seek for any tissue biomarker to predict responsiveness or resistance to the combination under study. We will explore changes in serum VEGF and other angiogenesis cytokine markers before and after initiation of ZD6474. See Appendix C.

3 STUDY SPECIFICATIONS

3.1 STUDY DESIGN

This is a Phase 1 trial in patients with previously treated stage IV colorectal cancer. The goal is to determine the maximum tolerated dose of ZD6474 with cetuximab and then the maximum tolerated dose of ZD6474, cetuximab and irinotecan.

During the dose finding phase, patients will be enrolled in cohorts of 3. All patients will receive standard doses of cetuximab (400 mg/m2 loading followed by weekly 250 mg/m2). Dose levels will be defined as such:

Dose levels	ZD6474 (daily)	Irinotecan (q2 wks)	Cetuximab † (weekly)
1	100 mg	--	250 mg/m2
2	100 mg	180 mg/m2	250 mg/m2
3	200 mg	180 mg/m2	250 mg/m2
4	300 mg	180 mg/m2	250 mg/m2

† - after 400 mg/m2 loading dose

The study will initially enroll into dose level 1. If 0 of 3 patients have a dose limiting toxicity (DLT) then we will advance to dose level 2. If 1 of 3 patients have a DLT, then 3 additional patients will be treated at dose level 1. If 2 patients have a DLT at

dose level 1, the study will close. If <33% of patients have a DLT at dose level 1, we will proceed to dose level 2.

At dose levels 2, we will initiate treatment with irinotecan. Treatment at dose levels 2-4 will begin with 2 weeks of only ZD6474 and Cetuximab. Irinotecan will begin on day 15 (week 3). If 0 of 3 patients have a DLT, we will proceed to the next dose level. If 1 of 3 patients have a DLT, then 3 additional patients will treated at the same dose level. If 2 patients have a DLT, then the MTD will be current dose level –1 (if on dose level 1, study closes). If < 33% of patients have a DLT, then the study will continue to the next dose level (if at level 4, the MTD for ZD6474 will be considered 300 mg).

Cycles of therapy will be defined as 4 weeks on therapy, even if drugs are held for a portion of that time.

3.2 DOSE LIMITING TOXICITIES

DLTs (defined as within the first 28 days of treatment in dose level 1and first 35 days in dose levels 2-4) will include: grade IV hematological toxicity > 7 days; fever and neutropenia; grade III diarrhea leading to hospitalization or lasting > 48 hours despite aggressive anti-diarrheal medication; grade IV diarrhea despite aggressive anti-diarrheal medication; grade IV vomiting despite optimal antiemetics; or grade III or higher nonhematological toxicity (excluding nausea, vomiting, diarrhea, or alopecia) lasting > 1 week; grade 4 skin toxicity; grade 3 or greater cardiac toxicities; or death.

If patients in dose levels 2-4 who have a DLT in the first 14 days (prior to initiation of irinotecan), the patient will not be counted towards the DLTs of that cohort and an additional patient will be substituted. For example, if one of the first 3 patients in dose level 2 have a DLT, that patient will not be calculated into the DLT tabulation for ZD6474, cetuximab and irinotecan and an additional patient will be substitute in that spot. If this scenario occurs twice, the study will be held and the study team will reevaluate the ZD6474 / cetuximab toxicities.

3.3 EXPECTED NUMBER OF PATIENTS

The number of patients expected to enroll would be 3-34 (dependent on dose level obtained and whether an MTD will be tested).

3.4 METHOD OF TREATMENT ALLOCATION

A patient number will be assigned by the Quality Assurance Office for Clinical Trials (QACT) to each patient upon registration. The overall study coordinator (based at DFCI) will assign the dose level.

3.5 DURATION OF THERAPY

Patients will continue the study treatment until disease progression, unacceptable toxicity, or development of any of the criteria for patient discontinuation as described in Section 11 of the protocol.

Patients will be considered to be on-study for the duration of their treatment and during the 30 days following treatment discontinuation. Treatment discontinuation is defined as the last day of study treatment.

All included patients will be followed up until recovery or stabilization of all adverse events or return to baseline condition.

4 STUDY POPULATION

4.1 INCLUSION CRITERIA

Patients must meet all of the following criteria in order to be eligible for entry into the trial:

(1) Eligible patients must have histologically or cytologically documented locally advanced or metastatic colorectal cancer

(2) 1-2 prior therapies for metastatic colorectal cancer. Prior adjuvant therapy not included in the number of priors unless recurrence within 12 months of last dose of therapy and then the adjuvant therapy will be counted as 1 prior.

(3) Patients must have measurable disease according to the RECIST criteria.

(4) Tumor is K-ras wildtype by method of choice at respective institution (testing codons 12 and 13). Since testing for K-ras should be standard of care for use of cetuximab in metastatic colorectal cancer, testing can be performed prior to signing consent or after consent but prior to enrollment.

(5) Age > 18 years

(6) ECOG Performance Score of 0-2.

(7) Patients should have completed any major surgery 4 weeks from registration. Patients must have completed any minor surgery 2 weeks from registration. Patients must have fully recovered from the procedure and have completely healed surgical incisions before starting therapy.. (Insertion of a vascular access device is not considered major or minor surgery.)

(8) Adequate bone marrow as evidenced by:
- Absolute neutrophil count ≥ 1,200/mL
- Platelet count ≥ 100,000/mL

(9) Adequate renal function as evidenced by serum creatinine ≤1.5 x ULRR or creatinine clearance ≥50 mL/minute (calculated by Cockcroft-Gault formula)

(10) Adequate hepatic function as evidenced by:
- Serum total bilirubin <= 1.5 mg/dL
- Alkaline phosphatase < 3X the ULN for the reference lab (< 5X the ULN for patients with known hepatic metastases)
- SGOT/SGPT < 3X the ULN for the reference lab (< 5X the ULN for patients with known hepatic metastases)

(11) Normal range of serum calcium (ionized or adjusted for albumin) and magnesium (supplementation permitted)

(12) Patients or their legal representatives must be able to read, understand and provide informed consent to participate in the trial.

(13) Patients of childbearing potential and their partners must agree to use an effective form of contraception during the study and for 90 days following the last dose of study medication (an effective form of contraception is an oral contraceptive or a double barrier method)

(14) For women who have not had a hysterectomy and under 60 years of age, a negative urine or serum pregnancy test. Male patients must be surgically

sterile or using an acceptable method of contraception during their participation in this study

4.2 EXCLUSION CRITERIA

Patients meeting any of the following criteria are ineligible for study entry:

(1) History of prior malignancy within the past 3 years except for curatively treated basal cell carcinoma of the skin, cervical intra-epithelial neoplasia, or treated localized prostate cancer with a current PSA of < 1.0 mg/dL on 2 successive evaluations, at least 3 months apart, with the most recent evaluation no more than 4 weeks prior to entry.

(2) Prior therapy with gefitinib, erlotinib, cetuximab, ABX-EGF or other specific EGFR inhibitor. Note that prior therapy with either bevacizumab and/or irinotecan is NOT an exclusion criteria

(3) Patients with known hypersensitivity to any of the components of cetuximab or ZD6474 or irinotecan

(4) Patients who are pregnant or lactating

(5) Any other medical condition, including mental illness or substance abuse, deemed by the clinician to be likely to interfere with a patient's ability to sign informed consent, cooperate and participate in the study, or interfere with the interpretation of the results.

(6) Potassium <4.0 mEq/L despite supplementation;

(7) Serum calcium (ionized or adjusted for albumin,) or magnesium out of normal range despite supplementation.

(8) Evidence of severe or uncontrolled systemic disease or any concurrent condition which in the Investigator's opinion makes it undesirable for the patient to participate in the trial or which would jeopardize compliance with the protocol.

(9) Clinically significant cardiac event such as myocardial infarction; New York Heart Association (NYHA) classification of heart disease $\geq$ 2 that, in the opinion of the Investigator, increases the risk of ventricular arrhythmia (see Appendix B) within 3 months before entry; or presence of cardiac disease

(10) History of arrhythmia (multifocal premature ventricular contractions (PVCs), bigeminy, trigeminy, ventricular tachycardia, or uncontrolled atrial fibrillation) which is symptomatic or requires treatment (CTCAE grade 3) or asymptomatic sustained ventricular tachycardia. Atrial fibrillation, controlled on medication is not excluded.

(11) Previous history of QTc prolongation as a result from other medication that required discontinuation of that medication.

(12) Congenital long QT syndrome,or 1st degree relative with unexplained sudden death under 40 years of age.

(13) Presence of left bundle branch block (LBBB.)

(14) QTc with Bazett's correction that is unmeasurable, or ≥480 msec on screening ECG. If a patient has QTc ≥480 msec on screening ECG, the screen ECG may be repeated twice. The average QTc from the three screening ECGs must be <480 msec in order for the patient to be eligible for the study.) Patients who are receiving a drug that has a risk of inducing

Torsades de Pointes are excluded if QTc is ≥460 msec (see Appendix A, Drug List 1; section 5.2)

(15) Any concomitant medication that are potent inducers of CYP3A4 (rifampicin, rifabutin, phenytoin, carbamazepine, Phenobarbital, St. John's Wort) For a comprehensive list of medications which may affect activity of CYP3A4, please visit:
http://www.medicine.iupui.edu/clinpharm/ddis/ClinicalTable.asp and http://www.pharmacytimes.com/issue/pharmacy/2008/2008-09/2008-09-8687.

(16) Hypertension not controlled by medical therapy (systolic blood pressure greater than 160 mm Hg or diastolic blood pressure greater than 100 mm Hg)

(17) Patients lacking physical integrity of the upper gastrointestinal tract or who have malabsorption syndrome.

(18) Currently active diarrhea that may affect patient's ability to absorb ZD6474 or tolerate potential diarrhea from study drugs.

(19) Receipt of any investigational agents within 30 days prior to commencing study treatment

(20) Last dose of prior chemotherapy or radiation therapy discontinued less than 4 weeks before the start of study therapy

(21) Incompletely healed surgical incisions, at the discretion of the investigator.

(22) Any unresolved toxicity greater than CTC grade 1 from previous anti-cancer therapy

(23) EGFR by immunohistochemistry will NOT be an exclusion

4.3 RESTRICTIONS

(1) Due to the experimental nature of ZD6474, female patients must be 1 year post-menopausal, surgically sterile or using acceptable method of contraception. Male patinets must be surgical sterile or using an acceptable method of contraception during their participations in this study and for 3 months after discontinuation of study drug.

5 STUDY PROCEDURES

5.1 TREATMENT REGIMEN

Treatment will be administered on an outpatient basis. Expected adverse events and appropriate dose modifications for study drugs are described in Section 6 and 7. No investigational or commercial agents or therapies other than those described below may be administered with the intent to treat the patient's malignancy.

This is a phase I study. Entry into dose levels are described in section 3. Initiation of ZD6474 will occur on the first day of treatment in the clinic or infusion unit. The patient will take the first dose of medication in the presence of the treating clinician or nurse. Thereafter, ZD6474 will be self-administered around the same time daily. If the patient inadvertently does not take the dose at their usual time, or has emesis within 30 minutes of taking ZD6474 tablet, the patient may take that dose any time

within 6 hours of the same day. The daily treatment schedule will be resumed the next day with the patient taking the scheduled dose at their usual time.

Cetuximab will be initiated on the first day of treatment at all dose levels. For the first treatment, ZD6474 should be given first and then at least 30 minutes should elapse prior to start of infusion of cetuximab. For the first treatment, patients will be premedicated with 50 mg IV of diphenhydramine 30-60 minutes prior to receiving 400mg/m^2 IV of Cetuximab over 120 minutes. Subsequently doses will be weekly infusions of 250 mg/m^2 IV over 60 minutes. The infusion rate of cetuximab must never exceed 10 mg/minute (5 mL/min). Premedication with diphenhydramine may be administered prior to subsequent doses, but at the Investigator's discretion, the dose of diphenhydramine (or similar agent) may be reduced.

In dose levels 2-4 and at the MTD (if includes irinotecan), irinotecan will be initiated on day 15 after administration of cetuximab. Irinotecan will be an IV infusion over 90 minutes every other week.

5.2 SUPPORTIVE THERAPIES

All occurrences of late-onset diarrhea should be treated aggressively with loperamide (imodium) or atropine sulfate / diphenoxylate hydrochloride (lomotil; lomotil can be substitute for loperamide in all of the below sections). Since uncontrolled diarrhea may lead to hospitalization for dehydration, patients should be instructed to maintain proper hydration. If warranted, patients should be given supplemental fluids (intravenously, if indicated) at any signs of dehydration (clinical signs plus weight loss of ≥ 1 kg at a particular weekly visit).

For patients entered into dose levels 2-4, atropine per institutional standards, may be given to treat acute-onset diarrhea from irinotecan. All patients will be instructed to begin taking loperamide at the earliest signs of diarrhea (i.e., first poorly formed or loose stool, first episode of an increase of two or more bowel movements in one day). Loperamide should be taken in the following manner: 4 mg at the first onset of diarrhea, then 2 mg every 2 hours around-the-clock until diarrhea-free for at least 12 hours. Patients may take loperamide 4 mg every 4 hours during the night. If patients require more than 16 mg of loperamide per day, alternative antidiarrheal medications should be institution with consideration of evaluation of the patient.

Antiemetics should be prescribed by the treating physician as clinically indicated if a patient develops nausea and/or vomiting. Prophylaxic antiemetics prior to IV therapies will be determined by treating clinicians.

Prophylactic use of a colony-stimulating factor (G-CSF or GM-CSF) is permitted after a first event of grade 3 or 4 neutropenia, as discretion of treating clinician. Use of erythropoietin (including Procrit and Aranesp) for treatment or disease-related anemia is permitted.

Supplementation of electrolytes (potassium, magnesium) is permitted and recommended to maintain serum potassium at least 3.0 mmol/L and serum magnesium at least 1.8 mg/dL or within institutional normal limits. Study agents may be administered while repletion occurs.

There is no standard, known, or established treatment proven effective for drug-related skin rashes or changes due to cetuximab or ZD6474. Most commonly, a pustular rash has been observed, which frequently improves with same dose of uninterrupted EGFR inhibitor therapy. The need for oral or topical antibiotics is a clinical decision of the investigator and should be preceded by a culture of affected areas and, if indicated, a dermatology consultation (though oral antibiotics in Appendix A are prohibited, topical antibiotics [even listed in Appendix A] are permitted). Systemic or topical steroids are discouraged for the treatment of skin toxicities. Treatment with topical erythromycin, topical tetracycline, topical clindamycin, topical silver sulfadiazine, and diphenhydramine is allowed at the discretion of the treating clinician.

The administration of radiation is not permitted and patients requiring radiotherapy will be removed from the study.

Concomitant use of the known potent inducers of CYP3A4: rifampicin, phenytoin, carbamazepine, barbiturates and St. John's Wort are not allowed within 2 weeks of study or during the study.

Concomitant use of medications generally accepted as having a risk of causing Torsades de Pointes (see Appendix A, List 1) are allowed at the time of study entry (if there is no appropriate alternate medication). Patients who are receiving drugs that may induce Torsades de Pointes, must have an additional ECG obtained 4-8 hours after the first dose of ZD6474.

Screening QTc must be <480 msec, or ≤460msec for patients taking medications with known risk of Torsades de Pointes.

Baseline QTc (Bazett's) will be determined by the average of no less than 3 consecutive ECG's (within 5-10 minutes of each other).

ECG's will be repeated at weeks 1, 2, 4, 8 and 12; at discontinuation; and as clinically indicated.

If QTc prolongation occurs at one of the usual assessment times, or at any other time, the QTc will be re-evaluated with no less than 3 consecutive ECG's (within 5-10 minutes) of one another.

6 DRUG FORMULATIONS AND SAFETY

6.1 ZD6474 (ZACTIMA)

Availability

ZD6474 will be supplied by AstraZeneca free of charge. AstraZeneca will pack tablets into high-density polyethylene (HDPE) bottles with child-resistant tamper-evident closures. Each bottle will be labeled with the statement: "Caution: New Drug - Limited by Federal (or USA) Law to Investigational Use". Instructions stating that the tablets are to be taken orally "as directed by your doctor" will be included. Information on the label will indicate the identity and quantity of tablets and storage conditions. Additional subject information will be identified as dictated by the protocol. The tablets should be stored in the original pack until use. For further information, investigators should refer to the investigational product label.

Tablets containing 100 mg of ZD6474 (ZACTIMA) are available and will be dispensed in a bottle containing 28 tablets, a quantity sufficient for 4 consecutive weeks of dosing.

Accountability

The trial treatment (ZD6474) must be used only as directed in the protocol. Records of overall dispensing and returns will be maintained separate from the case report forms (CRFs) recording the treatment dispensed to individual patients. Patients must return all unused medication and empty containers. These will be retained by study staff until collected by AstraZeneca authorized personnel.

The investigator must maintain accurate records accounting for the receipt of the investigational products (Astra Zeneca provides a copy of the investigational product shipping order for this purpose) and for the disposition of the material. This record keeping consists of a dispensing report including the identification of the person to whom the drug was dispensed, the quantity and date of dispensing and any unused drug returned on the CRFs.

The site should destroy unused study drug. Documentation indicating study drug was destroyed will be sent to AstraZeneca.

Certificates of delivery must be signed and dated. At the end of the trial, it will be necessary to reconcile delivery records with those of usage and returned stocks. Account must be given of any discrepancies.

Toxicities

The most common AEs associated with ZD6474 in the phase I and other monotherapy studies included rash, diarrhea and asymptomatic QTc prolongation. In Study ZD6474IL/0003 (the largest monotherapy ZD6474 study conducted as of this

date) the most frequent AEs for ZD6474 were diarrhea (55.4%), fatigue (36.1%), rash (27.7%), and nausea (24.1%). Approximately 10% of subjects who received ZD6474 had an adverse event of hypertension (HTN). The majority were Common Toxicity Criteria (CTC) grade 1 or 2. Three subjects developed CTC grade 3 HTN, and none were CTC grade 4. There were no serious adverse events (SAEs) of HTN. The median increase in systolic blood pressure for subjects who received ZD6474 was 10 mmHg; the median increase in diastolic blood pressure was 6 mmHg.

The cardiac events included a variety of terms without any apparent pattern. Respiratory events were primarily those that would be anticipated in subjects with advanced lung cancer. Three subjects receiving ZD6474 developed pulmonary embolism and 3 subjects developed interstitial lung disease, but cases were confounded by such factors as smoking, reduced mobility, infection, lung cancer progression and previous chemotherapy or radiation therapy. One subject in each arm developed a serious skin disorder. One subject who received ZD6474 developed a hematologic event, as did 2 subjects who received GEFITINIB. No subjects who received ZD6474 developed serious hepatotoxicity.

In Study 6474IL/0006, patients with advanced or metastatic NSCLC were enrolled after failure of prior platinum-based chemotherapy. Patients were randomized to treatment with a standard dose of Docetaxel and either placebo or 100 mg of ZD6474 or 300 mg of ZD6474. The most frequent AEs observed in this study were similar to those observed in prior trials for ZD6474 or reported for Docetaxel in the literature. The most common AEs and their frequencies as reported in the 300 mg ZD6474, 100 mg ZD6474 and placebo arms, respectively, were diarrhea (50.0%, 38.1%, 24.4%), fatigue (45.5%, 40.5%, 26.8%), neutropenia (31.8%, 26.2%, 19.5%) and nausea (29.5%, 26.2%, 19.5%). Rash was observed in 15.9%, 16.7% and 9.8% of patients in the three arms.

Gastrointestinal events (77.3%, 59.5%, 41.5%), and skin events (72.7%, 64.3%, 41.5%) were more common in the ZD6474-containing arms. For all arms, the majority of these events were common toxicity criteria (CTC) grade 1 and 2; however CTC grade 3/4 events were more prominent in the ZD6474 300 mg arm. Cardiac disorders occurred more frequently in ZD6474-containing arms (15.9%, 14.3%, 2.4%.). The majority were CTC grade 1/2 and included a variety of terms, none ventricular. The frequency of respiratory events (50.0%, 57.1%, 46.3%) was similar in all arms. Neutropenia (31.8%, 26.2%, 19.5%) and related terms were more common in ZD6474-containing arms, but this did not result in an increase in infection. There was little difference in frequency of other hematologic events.

Approximately 10% of patients receiving ZD6474 developed an AE of hypertension. The majority of events were CTC grade 1 or 2, and no events were CTC grade 4. There were no serious adverse events (SAEs) of hypertension. The maximum median rise in both systolic and diastolic blood pressure was approximately 8 mm Hg in patients who received ZD6474 300 mg, and approximately 4 mm Hg in patients who received ZD6474 100 mg.

There were 12 subjects with confirmed QTc prolongation in Study ZD6474IL/0003. Of these, 6 occurred in the first 28 days, and 2 in the following 28 days. The remaining 4 occurred sporadically, with the longest time to occurrence 323 days. There were 3 events of CTC grade 1 reversible dizziness in subjects with a confirmed QTc prolongation occurring within the first 4 weeks. Subjects with dizziness had other events that might have caused dizziness and the events were not well correlated in time with the actual QTc prolongation. There were no other potentially relevant adverse events in subjects with confirmed QTc prolongation within the first 4 weeks, and no relevant adverse events in subjects whose first confirmed QTc prolongation occurred more than 4 weeks after randomization.

In Study ZD6474IL/0006, 7 patients had confirmed QTc prolongation. Five (16%) occurred on the ZD6474 300 mg/Docetaxel arm, 4 of which occurred in the first 28 days, and 1 at day 70. Two (12%) occurred on the ZD6474 100 mg /Docetaxel arm, at days 22 and 43. No patients with confirmed QTc prolongation in study 6474IL/0006 experienced a potentially relevant AE. One patient in the run-in phase of trial 6 had received ZD6474 300 mg plus Docetaxel, was hospitalized with post-obstructive pneumonia as well as hypokalemia resulting from Prednisone and Fluorinef given to treat adrenal insufficiency resulting from adrenal metastases. He was noted to have a QTc interval of 626 millisecond (msec.) During this hospitalization he developed a 12 beat run of ventricular tachycardia, which was asymptomatic and resolved without treatment.

6.2 IRINOTECAN

Availability/Product Description

Irinotecan hydrochloride trihydrate [CPT-11, (4S)-4,11-diethyl-4-hydroxy-9-[(4-piperidino- pipeidino)carbonyloxyl-1H-pyrano[3',4':6,7]indolozino[1,2-b]quinoline-3,14(4H,12H) dinone hydrochloride trihydrate] (Camptosar®) is supplied in two forms: 2 ml vials containing 40 mg of drug and 5 ml vials containing 100 mg of drug. The drug is supplied in amber vials and appears as a pale yellow to yellow crystalline powder and pale yellow transparent solution when reconstituted. Irinotecan will be used from commercial stock.

Drug Administration

Irinotecan will be diluted and administered as per institutional standard and infused intravenously as per institutional standard. It is strongly recommended that irinotecan be given over 90 minutes after cetuximab infusion.

Storage and Stability

Irinotecan vials must be stored in a cool dry place, protected from light. Irinotecan is a relatively stable agent against heat and light but becomes slightly unstable against light in aqueous solution. Undiluted drug is stable for at least 3 years at room temperature. Irinotecan is stable for at least 24 hours in glass bottles or plastic bags when mixed with normal saline or 5% dextrose.

Toxicities in Humans

Diarrhea and neutropenia are the major dose-limiting toxicities of irinotecan. Diarrhea can occur in two situations, within the first 24 hours and after 2-4 days. In the former situation, diarrhea may be associated with abdominal cramping and is thought to be mediated by non-competitive inhibition of acetyl cholinesterase activity by irinotecan. This is usually readily treatable with atropine, 0.25-1 mg, intravenously.

When diarrhea occurs late (after 2-4 days), and often after the second or third weekly dose of irinotecan, it can be a potentially serious event. In contrast to early diarrhea, delayed diarrhea appears to be secretory in nature and to result from abnormal intestinal ion transport. Anti-diarrheal agents, such as loperamide, diphenoxylate-atropine (Lomotil®), octreotide, scopolamine and bismuth are typically ineffective once grade IV diarrhea has occurred. The diarrhea usually lasts 5-7 days before resolving. Early recognition of diarrhea and prompt institution of an intensive and prolonged course of loperamide appears to be the most effective approach to this problem. It is recommended that patients take 4mg of loperamide immediately after their first loose bowel movement. Loperamide 2mg should be taken every 2 hours for a total of 12 hours if diarrhea persists. If no further loose bowel movements occur, therapy may be discontinued.

Myelosuppression is manifested primarily as leukopenia and neutropenia. Other toxicities include nausea, vomiting, alopecia and cumulative fatigue or asthenia. Instances of possible drug related hepatic toxicity have occurred, but are rare. Further descriptions are found in the package insert for irinotecan.

Several cases of interstitial pneumonitis have been reported, predominantly in the Japanese literature, with an incidence of 1.3%. Most reports have occurred in patients with primary lung tumors, where it is unclear whether the pulmonary toxicity was attributable to irinotecan, to progression of the underlying malignancy or to a combination effect. Clinically, this is manifested as worsening shortness of breath, fever and a reticulonodular infiltrate on CXR. Corticosteriods may be helpful in treatment. Hence, the exact mechanism by which pulmonary toxicity is mediated is unknown; however, a current belief is that it may represent an idiosyncratic reaction to the drug. No characteristic histologic correlates have been identified.

6.3 CETUXIMAB

Availability/Product Description
Cetuximab is an anti-EGFR receptor humane-to-murine chimeric antibody. Cetuximab is expressed in SP2/0 myeloma cell line, grown in large scale cell culture bioreactors and purified to a high level purity using several purification steps including protein A chromatography, ion exchange chromatography, low pH treatment and nonofiltration. Cetuximab is not known to be a vesicant.

The product is formulated to 2 mg protein/mL with phosphate buffered saline, pH 7.2 ± 0.2 and aseptically filled into sterile glass vials, 100 mg per 50 cc vial, and stored as a liquid at 2 to 8 °C. Each vial contains the following active and inactive ingredients per 1.0 ml: 2 mg of cetuximab, 145 nmol/L sodium chloride, and 10 mmol/L sodium phosphate.

Cetuximab is obtained in single-use, ready-to-use 50-mL vials containing 2 mg/mL of product. Cetuximab must be stored under refrigeration at +2˚C to +8˚C (+36˚F to +46˚F). DO NOT FREEZE CETUXIMAB. Commercially available cetuximab will be used in this protocol and charged to patient’s insurance company.

Drug Administration
Cetuximab requires no dilution. Patients will receive weekly cetuximab intravenous infusions via either gravity drip, infusion pump, or syringe pump with in-line filtration. Cetuximab should not be mixed with or diluted with other drugs or solutions for infusion . The dose and volume of the study drug to be infused are dependent upon the patient’s BSA. The infusion rate must never exceed 10 mg/minute (5 mL/minute).

Appropriate mask, protective clothing, eye protection, gloves and Class II vertical-laminar-airflow safety cabinets are recommended during preparation and handling. Opened vials must be disposed of as chemotherapy or biohazardous waste provided documented procedures for destruction are in place.

Patients no longer require a ‘test dose’ prior to the loading dose of Cetuximab.

For the first treatment with cetuximab, patients will be premedicated with 50 mg IV of Benadryl 30-60 minutes prior to receiving 400mg/m^2 of Cetuximab. Premedication may be administered prior to subsequent doses, but at the Investigator’s discretion, the dose of diphenhydramine (or similar agent) may be reduced.

The initial dose of cetuximab is 400 mg/m^2 intravenously administered over 120 minutes, followed by weekly infusions at 250 mg/m^2 IV over 60 minutes. **The infusion rate of cetuximab must never exceed 10 mg/minute (5 mL/min).**

Vital signs will be obtained per institute standards, and as necessary to monitor for Cetuximab-related adverse events during and post infusion.
CAUTION: Hypersensitivity reactions, including severe reactions, may occur during or following cetuximab administration. Most hypersensitivity reactions occur with the first infusion of cetuximab, but some patients' first hypersensitivity reactions have been reported following subsequent doses (a severe reaction occurred in one patient following the 8th dose). A nurse must be present in the immediate treatment area throughout the infusion and observation period. A physician must be in close proximity to the patient treatment area. **Patients should be instructed to report any delayed reactions to the Investigator immediately.**

Severe hypersensitivity reactions, characterized by airway obstruction (bronchospasm, stridor and hoarseness, urticaria, and/or hypotension, require immediate interruption of cetuximab infusion and permanent discontinuation from further treatment with cetuximab. Appropriate medical therapy including epinephrine, corticosteroids, diphenhydramine, bronchodilators, and oxygen should be available for use in the treatment of such reactions. Subjects should be carefully observed until the complete resolution of all signs and symptoms.

Mild to moderate (Grade 1 or 2) hypersensitivity reactions are managed by slowing the infusion rate for cetuximab and by employing prophylactic use of antihistamine medications for subsequent doses. For mild or moderate hypersensitivity reactions manifesting only as delayed drug fever, maintain the cetuximab dose and infusion rate. Administration of acetaminophen or a non-steroidal anti-inflammatory drug (NSAID) may be administered prior to subsequent cetuximab infusions, if not otherwise contraindicated in subjects.

Once a cetuximab infusion rate has been decreased due to a hypersensitivity reaction, it will remain decreased for all subsequent infusions. If a subject has a second hypersensitivity reaction with the slower infusion rate, the infusion should be stopped and the subject should receive no further cetuximab treatment.

Once a cetuximab infusion rate has been decreased due to an allergic/hypersensitivity reaction, it will remain decreased for all subsequent infusions. If the patient has a second allergic/hypersensitivity reaction with the slower infusion rate, the infusion should be stopped, and the patient should receive no further cetuximab treatment. If a patient experiences a Grade 3 or 4 allergic/hypersensitivity reaction at any time, the patient should receive no further cetuximab treatment. If there is any question as to whether an observed reaction is an allergic/hypersensitivity reaction of Grades 1 - 4, the overall principal investigator should be contacted immediately to discuss and grade the reaction.

Toxicities in Humans

The incidence of the most significant or common adverse events occurring in single-agent cetuximab trials and by relationship to cetuximab, are presented in the following table:

Adverse Events in Single-Agent Cetuximab Trials*
(n = 281, as of November 30, 2002)

	All Adverse Events				Related Adverse Events**			
	Grades 1 - 4		Grades 3 & 4		Grades 1 - 4		Grades 3 & 4	
	N	(%)	N	(%)	N	(%)	N	(%)
Asthenia	127	(45)	21	(7)	71	(25)	8	(3)
Nausea	76	(27)	3	(1)	38	(14)	0	(0)
Rash	87	(31)	8	(3)	86	(31)	6	(2)
Acne	104	(37)	20	(7)	104	(37)	20	(7)
Diarrhea	53	(19)	3	(1)	26	(9)	3	(1)
Vomiting	66	(24)	9	(3)	26	(9)	2	(1)
Fever	99	(35)	2	(1)	75	(27)	0	(0)
Anorexia	53	(19)	8	(3)	13	(5)	1	(< 1)
Weight Loss	24	(9)	0	(0)	3	(1)	0	(0)
Dry Skin	35	(13)	2	(1)	33	(12)	2	(1)
Dyspnea	51	(18)	20	(7)	11	(4)	3	(1)
Mucous Membrane Disorder	17	(6)	3	(1)	9	(3)	1	(< 1)
Headache	64	(23)	3	(1)	40	(14)	2	(1)
Stomatitis	26	(9)	7	(3)	13	(5)	0	(0)
Chills	13	(5)	2	(1)	0	(0)	0	(0)
Pruritus	25	(9)	2	(1)	21	(8)	1	(< 1)
Nail Disorder	21	(8)	0	(0)	18	(6)	0	(0)
Allergic Reaction	24	(9)	8	(3)	18	(6)	8	(3)
Anaphylactoid Reaction	4	(1)	4	(1)	4	(1)	4	(1)

* Data from 281 patients enrolled in 12 trials receiving single-agent cetuximab. All adverse events reported by investigator and by relationship to cetuximab.
** Possible, probably or definite relationship to cetuximab as reported by investigator.

The most common adverse event associated with cetuximab administration is acne-like rash. Acne-like rash usually occurs on the face, upper chest, and back, but occasionally extends to the extremities and is characterized by multiple follicular-or pustular-appearing lesions characterized histologically as lymphocytic perifolliculitis or suppurative superficial folliculitis in patients with metastatic carcinoma. The onset of the rash is generally within the first 3 weeks of therapy. In patients who received cetuximab at doses less than 100 mg/m^2, acne-like rash was reported infrequently and was restricted to Grades 1 or 2. A number of therapeutic interventions have been attempted, including oral and topical antibiotics, topical steroids, and rarely, oral steroids. The value of these measures is unknown since definitive clinical trials have

not been performed. The etiology of the acne-like skin rash is believed to be the result of cetuximab binding to EGFR in the epidermis.

An uncommon adverse event reported is a nail disorder characterized as paronychial inflammation with associated swelling of the lateral nail folds of the toes and fingers. The most commonly affected digits are the great toes and thumbs. According to Investigators, the nail disorder may persist for up to 3 months after discontinuation of cetuximab. Preliminary analysis in patients treated at the doses to be administered in this trial (400 mg/m^2 initial dose, followed by 250 mg/m^2 weekly) revealed that incidence of nail disorder is greater in patients who received > 6 cetuximab infusions (~10%) compared with patients treated with ≤ 6 infusions (~3%).

As cetuximab is a protein, the potential exists for allergic reaction to occur during or following cetuximab administration. In clinical trials, severe hypersensitivity reactions (including allergic and anaphylactic reactions), characterized by the rapid onset of airway obstruction (bronchospasm, stridor, hoarseness), urticaria, and/or hypotension, have been observed in approximately 3% of patients treated with cetuximab. The large majority of these severe reactions occurred with the first infusion of cetuximab and were observed during or within one hour of the completion of dosing.

7 DOSE and SCHEDULE MODIFICATIONS

This Phase I study is intended to establish the maximum tolerated dose (MTD) of the combination of cetuximab, ZD6474 and irinotecan. DLTs (defined as within the first 28 days of treatment in dose level 1and first 35 days in dose levels 2-4) will include: grade IV hematological toxicity > 7 days; fever and neutropenia; grade III diarrhea leading to hospitalization or lasting > 48 hours despite aggressive anti-diarrheal medication; grade IV diarrhea despite aggressive anti-diarrheal medication; grade IV vomiting despite optimal antiemetics; or grade III or higher nonhematological toxicity (excluding nausea, vomiting, diarrhea, or alopecia) lasting > 1 week; grade 4 skin toxicity; grade 3 or greater cardiac toxicities; or death. If patients in dose levels 2-4 who have a DLT in the first 14 days (prior to initiation of irinotecan), the patient will not be counted towards the DLTs of that cohort and an additional patient will be substituted. For example, if one of the first 3 patients in dose level 3 have a DLT, that patient will not be calculated into the DLT tabulation for ZD6474, cetuximab and irinotecan and an additional patient will be substitute in that spot. If this scenario occurs twice, the study will be held and the study team will reevaluate the ZD6474 / cetuximab toxicities.

Patients should remain on the assigned doses of therapy throughout the period of assessment of DLTs (first 28 days in dose level 1and 35 days in dose levels 2-4) unless a DLT is observed or the patient is withdrawn from study for other reasons. The following guidelines for dosing delay and/or dose modifications apply to patients who either experience a DLT during the first 28 days (dose level 1) or 35 days (dose levels 2-4) OR develop treatment-related toxicities after that period of assessment.

7.1 ZD6474 DOSE MODIFICATIONS

For guidance on the management of QTc prolongation, see Figure 1 below. For all other toxicities, the dose of study treatment will be withheld for up to 3 weeks until the toxicity has resolved to CTCAE grade 1 or better, and then study treatment may be restarted. Patients will be withdrawn from the study if toxicity does not resolve to CTCAE grade 1 or better within 3 weeks. Dose reduction/re-challenge for each toxicity criterion will be managed as discussed in the sections that follow.

7.1.1 Dose reduction table

Patients will be withdrawn from the study if toxicity does not resolve to ≤ CTCAE grade 1 within 3 weeks. Dose reduction will be dependent on the original dose. A guideline for dose reduction is shown in the table below. There will be no dose reescalations for an individual patient (ie. dose reductions for ZD6474 will be permanent).

Current dose	Reduced dose	ZD6474 dispensed for reduced dose	Tablets per daily dose
300 mg	200 mg	100-mg ZD6474 tablets	2
200 mg	100 mg	100-mg ZD6474 tablets	1
100 mg	100 mg **every other day**		1 every other day

7.1.2 QTc PROLONGATION

Patients will have ECGs performed to monitor QTc interval (using Bazett's correction) as outlined in the study plan.

Figure 1 Flowchart detailing management of QTc prolongation

Figure 2 Flowchart detailing management of QTc prolongation

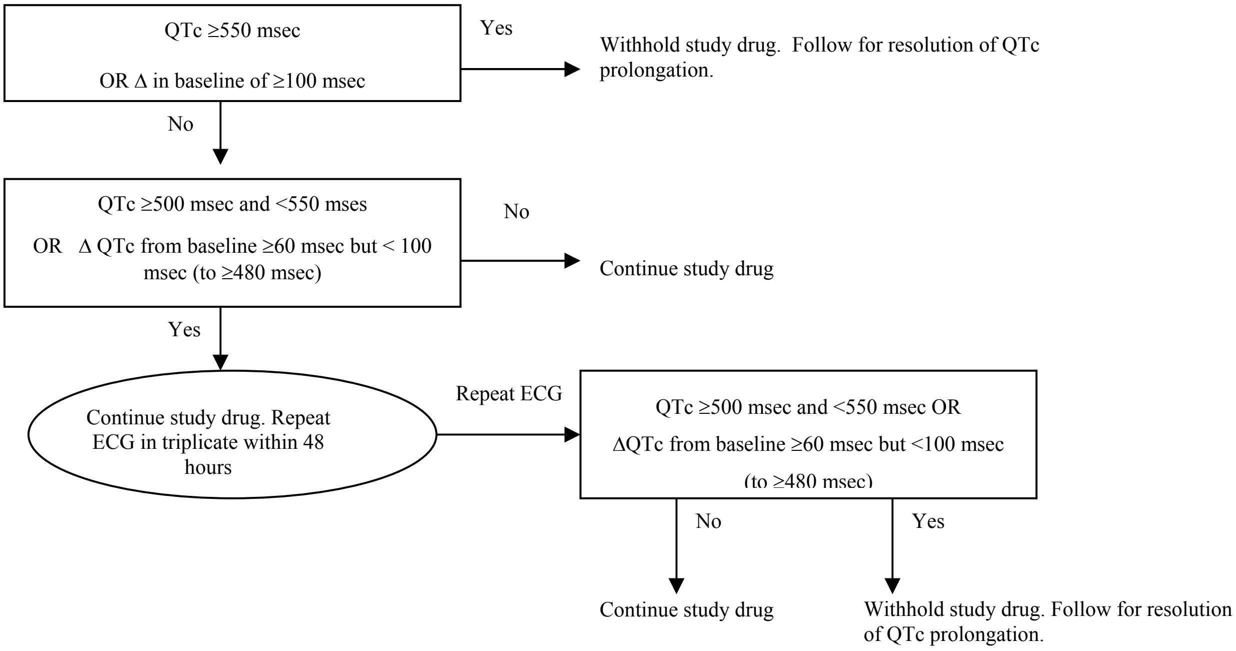

For this study, QTc prolongation is defined as:

(1) A single QTc value of ≥550 msec or an increase of ≥100 msec from baseline

OR

(2) Two consecutive ECG measurements, within 48 hours of one another, in which either of the following criteria are met for both QTc values (the second being the mean of 3 consecutive ECGs):

- A QTc interval ≥500 msec but <550 msec **OR**
- An increase of ≥60 msec, but <100 msec, from baseline QTc to a QTc value ≥480 msec.

Use baseline QTc as noted in the Required Data Table (Section 10, ‡)

7.1.3 Management of Patients With QTc Prolongation

For a single QTc value of ≥550 msec or an increase of ≥100 msec from baseline, ZD6474 must be withheld. ECGs and electrolytes should be followed 3 times a week until QTc falls below 480 msec or baseline, whichever is higher. ZD6474 treatment may be resumed at a lower dose after the QTc recovers to <480 msec or baseline. For a QTc interval ≥500 msec, but <550 msec, or an increase of ≥60 msec but <100 msec from baseline QTc to a QTc value ≥480 msec, ZD6474 may be continued but a repeat ECG (in triplicate) must be obtained within 48 hours. If QTc prolongation is confirmed, ZD6474 should be withheld. ECGs and electrolytes should be checked 3 times a week until QTc falls below 480 msec or baseline, whichever is higher. ZD6474 treatment may be resumed at a lower dose, as outlined in section 7.1.1 after the QTc recovers to <480 msec or baseline. If the patient does not meet the criteria for QTc prolongation at the repeat ECG, then the patient should continue treatment and resume the ECG schedule as outlined in the Study Plan.

If ZD6474 is restarted after the QTc prolongation has resolved, ECGs should be performed 1, 2, 4, 8, 12 weeks and then every 3 months after treatment is restarted. If ZD6474 must be withheld for >3 weeks to allow for QTc prolongation to recover to <480 msec or baseline, the patient will withdrawn from study. If QTc prolongation recurs after the dose reduction as detailed, the patient must permanently discontinue treatment with study medication

7.1.4 Management of Skin Toxicity

It is strongly recommended that all patients follow a program of sun protective measures while receiving study therapy and for 3-4 weeks after discontinuing study therapy. The aim is to reduce the risk of development of skin rash, minimize the severity of skin rash, and to minimize the requirement for dose reduction of study therapy.

If a patient develops a skin rash, the following actions are recommended to the Investigator for the management of this reaction:

- A variety of agents can be used to manage skin rashes. These include mild to moderate strength steroid creams, topical or systemic antibiotics, topical or systemic antihistamines, and occasionally retinoid creams.

- The rash should be graded/assessed by a physician as soon as possible according to the CTCAE cutaneous toxicity criteria and documented accordingly

- If a rash of CTCAE grade 2 or higher is detected, immediate symptomatic treatment should be provided.

- If a rash of CTCAE grade 3 is detected, ZD6474 should be withheld until recovery to grade 1 or below. ZD6474 should be dose reduced as per section 7.1.1.

- Grade 4 rash will result in study withdrawal

If ZD6474 must be withheld for >3 weeks due to cutaneous toxicity, the patient will be withdrawn from study.

7.1.5 Management of Gastrointestinal Toxicity

Nausea, vomiting, or both may be controlled with antiemetic therapy. Diarrhea should be treated with standard medications to avoid dose modification or interruption, if possible. Electrolyte supplementation with regular laboratory monitoring should be used, when appropriate, to maintain electrolytes within normal limits and prevent an increased risk of QTc prolongation. No dose modifications will be made because of grade 1 or 2 diarrhea. If grade 3 diarrhea develops, ZD6474 should be withheld until diarrhea resolves to grade 1 or below. Patients who are clinically unstable because of diarrhea or other intercurrent medical illness must be admitted and evaluated using telemetry, until clinically stable. Upon recovery, ZD474 may resume at a permanently reduced dose if patient developed diarrhea in the setting of optimal antidiarrheal management; else ZD6474 can be maintained at the same dose or reduced (at discretion of treating physician) and patient must be instructed as to optimal diarrhea management. If ZD6474 must be withheld for more than 3 weeks for resolution of diarrhea, the patient will withdrawn from study.

7.1.6 Management of Other Toxicity

If any other grade 3 or 4 toxicity that is not outlined elsewhere in Sections 7.1 develops and is deemed attributable to ZD6474, ZD6474 should be withheld until the toxicity resolves to grade 1 or below. Upon recovery, treatment may resume at a permanent reduced dose. If ZD6474 must be withheld for more than 3 weeks for resolution of toxicity, the patient will be withdrawn from study.

Patients who develop CTCAE grade 3 hypertension may continue on therapy if blood pressure is controlled on antihypertensive medication. If blood pressure cannot be stabilized with increased antihypertensive medication, study treatment must be discontinued and cannot be resumed until blood pressure is controlled to baseline level. Patients with CTCAE grade 4 hypertension should discontinue study treatment and cannot resume therapy until blood pressure is controlled to baseline level. If study treatment must be interrupted for more than 3 weeks to allow for toxicity to resolve, the patient’s study participation will be discontinued.

7.1.7 Summary of Guidance on the Management of Toxicity for ZD6474

Toxicity	ZACTIMA
QTc value ≥550 msec or prolonged ≥100 msec from baseline	Withhold dose; if QTc recovers to <480 msec or baseline, then reduce dose as per section 3.3.5.1. If QTc does not recover to <480 msec or baseline within 3 weeks, patient will permanently discontinue ZD6474.
QTc value ≥500 msec or prolonged ≥60 msec from baseline	Continue dosing; repeat ECG (in triplicate) within 48 hours; if repeat ECG meets criteria, withhold dose; then if QTc recovers to <480 msec or baseline, reduce dose as per section 3.3.5.1. If QTc does not recover to <480 msec or baseline within 3 weeks, patient must permanently discontinue treatment with study medication. Or, if the repeat ECG does not meet criteria, patient should continue study medication.
Grade 3 or 4 cutaneous	Withhold dose until toxicity has resolved to CTCAE grade 1 or less, then reduce dose as per section 7.1.4. If ZD6474 must be withheld for >3 weeks due to cutaneous toxicity, the patient will be withdrawn from study.
Other grade 3 or 4 toxicity related to ZD6474	Withhold dose until toxicity has resolved to CTCAE grade 1 or less, then reduce dose as per section 7.1.6. If study treatment must be interrupted for more than 3 weeks to allow for toxicity to resolve, the patient's study participation will be discontinued.

7.2 CETUXIMAB DOSE MODIFICATIONS

7.2.1 Management of Infusion Reactions

Infusion reactions should be graded according to allergic reaction/hypersensitivity. Caution must be exercised with every cetuximab infusion, as there were patients who experienced their first severe infusion reaction during later infusions. Severe infusion reactions require the immediate interruption of cetuximab therapy and permanent discontinuation from further treatment. Appropriate medical therapy including epinephrine, corticosteroids, intravenous antihistamines, bronchodilators, and oxygen should be available for use in the treatment of such reactions. Patients should be carefully observed until the complete resolution of all signs and symptoms. In clinical trials, mild to moderate infusion reactions were managed by slowing the infusion rate of cetuximab and by continued use of antihistamine premedications (eg, diphenhydramine) in subsequent doses. If the patient experiences a mild or moderate (Grade 1 or 2) infusion reaction, the **infusion rate** should be permanently reduced by 50%. For grade 1 or 2 reactions manifesting only as delayed drug fever, maintain the cetuximab dose and infusion rate. Consideration should be given to administration of acetaminophen or a nonsteroidal anti-inflammatory drug (NSAID)

prior to the subsequent cetuximab infusion if not contraindicated in subjects. Dose and schedule of these agents is entirely at the investigator's discretion.

Cetuximab should be immediately and permanently discontinued in patients who experience severe (Grade 3 or 4) infusion reactions and will need to be removed from the trial.

7.2.2 Treatment of Isolated Drug Fever

In the event of isolated drug fever, the investigator must use clinical judgment to determine if the fever is related to the study drug or to an infectious etiology. If a patient experiences isolated drug fever, for the next dose, pre treat with acetaminophen or non-steroidal anti-inflammatory agent (investigator discretion), repeat antipyretic dose 6 and 12 hours after cetuximab infusion. The infusion rate will remain unchanged for future doses. If a patient experiences recurrent isolated drug fever following premedication and post-dosing with an appropriate antipyretic, the infusion rate for subsequent dosing should be 50% of previous rate. If fever recurs following infusion rate change, the investigator should assess the patient's level of discomfort with the event and use clinical judgment to determine if the patient should receive further cetuximab.

7.2.3 Management of Pulmonary Toxicity

In the event of acute onset (grade ≥2) or worsening pulmonary symptoms which are not thought to be related to underlying cancer, cetuximab therapy should be interrupted and a prompt investigation of these symptoms should occur. Cetuximab retreatment should not occur until these symptoms have resolved to grade 1. If interstitial lung disease is confirmed, cetuximab should be discontinued and the patient should be treated appropriately and withdrawn from study.

7.2.4 Management of Dermatologic Toxicity

Patients developing dermatologic toxicities while receiving cetuximab should be monitored for the development of inflammatory or infectious sequelae, and appropriate treatment of these symptoms initiated. Since both ZD6474 and cetuximab can lead to dermatological toxicities, the following schema should be followed required dose reductions:

* For the first instance of grade 3, dose-reduce ZD6474 only.
* For subsequent instances of grade 3 rash, both ZD6474 and cetuximab should be dose reduced

Dose reductions of cetuximab are permanent; no re-escalation of dose is allowed. Cetuximab cannot be held for more than 3 weeks; if cetuximab is held for more than 3 weeks, the patient will need to be discontinued from the trial. Dose reductions of cetuximab are as follows:

Grade 3 Rash	Cetuximab	Dose modification
1st occurrence	Hold infusion until grade 1 or less	Continue at 250 mg/m2 *
2nd occurrence	Hold until grade 1 or less	Reduce to 200 mg/m2
3rd occurrence	Hold until grade 1 or less	Reduce to 150 mg/m2
4th occurrence	Discontinue study treatment	

* ZD6474 will be reduced for first occurrence

7.2.4 Management of Nail Toxicity

Cetuximab may be skipped for up to 3 weeks for severe nail / fingertip cracking. Followed above table (for skin toxicity), though ZD6474 will not be dose reduced at the first instance. Cetuximab will be reduced as in the Table for skin toxicity starting at the row for 2nd occurrence of rash.

7.3 Irinotecan Dose Reductions

Treatment with irinotecan can resume if ANC ≥ 1200/mm3, the platelet count is ≥ 100,000/mm3, and any other irinotecan-related toxicity resolved to ≤ grade 1. If irinotecan is held for irinotecan-related toxicities that are not attributable to ZD6474 or cetuximab, treatment with cetuximab and ZD6474 may continue. **Irinotecan cannot be delayed for more than 3 weeks or the patient should be removed from protocol therapy.**

7.3.1 Irinotecan Dose Levels

Current Irinotecan Dose	Dose reduction
180 mg/m^2	150 mg/m^2
150 mg/m^2	120 mg/m^2
120 mg/m^2	100 mg/m^2
100 mg/m^2	85 mg/m^2

Dose reductions beyond 85 mg/m^2 will not be permitted. Once the irinotecan dose is reduced, no dose escalation is permitted.

7.3.2 Hematological toxicities (excluding febrile neutropenia)

Dose reductions will be determined based on blood counts on the day irinotecan should be resumed (unless patient had documentation of grade 4 neutropenia OR had

an episode of febrile neutropenia defined as ANC < 1000 and temperature at least 38.5^{o}C). No dose reductions will be made for anemia.

Hematological Toxicity (on day of start of scheduled dose of irinotecan unless febrile neutropenia or grade 4 neutropenia)	*Action*	*Resumption of therapy*
ANC 1000-1199<br>Platelets 75-99 K	Hold therapy until ANC at least 1200 and Platelets at least 100,000	If resolution in 1 week, continue same dose *<br>If resolution > 1 week, then dose reduce 1 level
Grade 3 Neutropenia<br>Grade 2 - 4 Thrombocytopenia	Hold therapy until ANC at least 1200 and Platelets at least 100,000	Once resolution to ANC at least 1200 and Platelets at least 100,000, dose reduce 1 level for subsequent treatments *
Febrile neutropenia<br>Grade 4 Neutropenia<br>(at any time during therapy)	Hold therapy until resolution of acute episode and above hematological requirements met	Dose reduce 1 level for subsequent treatments *

* May consider growth factor support for subsequent treatments.

7.3.3 Cholinergic reactions

Lacrimation, rhinorrhea, miosis, diaphoresis, hot flashes, flushing, abdominal cramping, diarrhea, or other symptoms of early cholinergic syndrome may occur during or shortly after receiving irinotecan. Atropine, 0.25-1.0 mg IV or SC may be used to treat these symptoms. In patients with troublesome or recurrent symptoms, prophylactic administration of atropine shortly before irinotecan therapy may be considered. Additional antidiarrheal measures may be used at the discretion of the treating physician. Anticholinergics should be used with caution in patients with potential contraindications (e.g., obstructive uropathy, glaucoma, tachycardia, etc.).

7.3.4 Late diarrhea (e.g., developing more than 24 hours after irinotecan)

Late diarrhea should be managed with anti-diarrheal medications as judged by treating physician (including loperamide, lomotil, deodorized tincture of opium). Irinotecan must be held until diarrhea resolves to no greater than grade 1. The following dose modifications are based on toxicities experienced during a cycle:

Diarrhea grade	*Action*	*Resumption of therapy*
Grade 2/3 diarrhea<br>Not optimal antidiarrheal therapy	Hold until no greater than grade 1	Resume same dose but optimize anti-diarrheal management
Grade 2/3 with optimal antidiarrheal therapy	Hold until no greater than grade 1	Resume with 1 level dose reduction
Grade 4 diarrhea	Hold until no greater than grade 1	Resume with 1 level dose reduction

7.3.4 Nausea/Emesis

Nausea / emesis should be managed with anti-nausea medications as judged by treating physicial. Irinotecan must be held until nausea/emesis resolves to no greater than grade 1. The following dose modifications are based on toxicities experienced during a cycle:

Grade	*Action*	*Resumption of therapy*
Grade 2/3 nausea<br>Grade 2/3 vomiting<br>Not optimal antinausea meds	Hold until no greater than grade 1	Resume same dose but optimize anti-nausea management
Grade 2/3 nausea<br>Grade 2/3 vomiting with optimal antinausea meds	Hold until no greater than grade 1	Resume with 1 level dose reduction
Grade 4 vomiting	Hold until no greater than grade 1	Resume with 1 level dose reduction

7.3.5 Other toxicities

Other toxicities attributable to irinotecan as deemed by the investigator (except alopecia) should be managed as such

Grade	*Action*	*Resumption of therapy*
Grade 0,1,2 other toxicity	Continue irinotecan	No dose reductions
Grade 3/4 toxicity (except alopecia)	Hold until no greater than grade 2	Resume with 1 level dose reduction

8 ADVERSE EVENTS

8.1 Monitoring of Adverse Events

The definitions of Adverse Events (AEs) and Serious Adverse Events (SAEs) are given below. It is of the utmost importance that all staff involved in the study be familiar with the content of this section. The Principal Investigator is responsible for ensuring this.

8.2 Events Definitions

An **Adverse Event (AE)** is the development of an undesirable medical condition or the deterioration of a pre-existing medical condition following or during exposure to a pharmaceutical product, whether or not considered causally related to the product. An undesirable medical condition can be symptoms (eg, nausea, chest pain), signs (eg, tachycardia, enlarged liver) or the abnormal results of an investigation (eg, laboratory findings, ECG). In clinical studies, an AE can include an undesirable medical condition occurring at any time, including run-in or washout periods, even if no study treatment has been administered.

Any detrimental change in a patient's condition subsequent to them entering the study and during the follow-up period should be considered an AE. When there is a deterioration in the condition for which the study treatment is being used, there may be uncertainty as to whether this is lack of efficacy or an AE. In such cases, unless the reporting physician considers that study treatment contributed to the deterioration or local regulations state to the contrary, the deterioration should be considered a lack of efficacy. Signs and symptoms of disease progression are therefore not considered AEs.

The development of a new cancer should be regarded as an AE. New cancers are those that are not the primary reason for administration of study treatment and have been identified after inclusion of the patient into the clinical study.

A **Serious Adverse Event (SAE)** is an AE occurring during any study phase (ie, run-in, treatment, washout, follow-up), and at any dose of the investigational product, comparator or placebo, that fulfills one or more of the following criteria:

- Results in death
- Is immediately life-threatening
- Requires inpatient hospitalization or prolongation of existing hospitalization
- Results in persistent or significant disability or incapacity
- Is a congenital abnormality or birth defect
- Is an important medical event that may jeopardize the patient or may require medical intervention to prevent one of the outcomes listed above.

Any event or hospitalization that is unequivocally due to progression of disease, as determined by the investigator, must not be reported as an SAE, however should be communicated to AstraZeneca.

The causality of SAEs (their relationship to all study treatment) will be assessed by the investigator(s) and communicated to AstraZeneca. Causality is a determination of whether there is a reasonable possibility that the drug may have caused or contributed to an adverse event. It includes assessing temporal relationships dechallenge/rechallenge information, association (or lack of association) with underlying diseases, and the presence (or absence) or a lack of one or more likely causes.

The Investigator must attempt to determine if an adverse event is in some way related to the use of the study drug. This relationship should be described as follows:

Unlikely: The event is clearly due to causes distinct from the use of the study drug, such as a documented pre-existing condition, the effect of a concomitant medication, a new condition which, based on the pathophysiology of the condition, and the pharmacology of the study drug, would be unlikely related to the use of the study drug.

Possible: The event follows a reasonable temporal sequence from administration of the study drug or the event follows a known response pattern to the study drug BUT the event could have been produced by an intercurrent medical condition which, based on the pathophysiology of the condition, and the pharmacology of the study drug, would be unlikely related to the use of the study drug or the event could be the effect of a concomitant medication

Probable: The event follows a reasonable temporal sequence from administration of the study drug and the event follows a known response pattern to the study drug AND the event cannot have been reasonably explained by an intercurrent medical condition or the event cannot be the effect of a concomitant medication

Definite: The event follows a reasonable temporal sequence from administration of the study drug, the event follows a known response pattern to the study drug and based on the known pharmacology of the study drug, the event is clearly related to the effect of the study drug

Unknown: Based on the evidence available, causality cannot be ascribed

8.3 Recording and Reporting

8.3.1 Serious Adverse Events Reportable to the FDA

Investigators and other site personnel must inform the FDA, via a MedWatch form, of any serious or unexpected adverse events that occur in accordance with the reporting obligations of 21 CFR 312.32, and will concurrently forward all such reports to AZ. A copy of the MedWatch report must be faxed to AstraZeneca at the time the event is reported to the FDA. It is the responsibility of the investigator to compile all necessary information and ensure that the FDA receives a report according to the FDA reporting requirement timelines and to ensure that these reports are also submitted to AstraZeneca at the same time.

A cover page should accompany the MedWatch form indicating the following:

- ZD6474 Investigator Sponsored Study (ISS)
- The investigator IND number assigned by the FDA
- The investigator's name and address
- The trial name/title and AstraZeneca reference number

Investigative site must also indicate, either in the SAE report or the cover page, the causality of events in relation to all study medications and if the SAE is related to disease progression, as determined by the principal investigator.

Send SAE report and accompanying cover page by way of fax to AstraZeneca's designated fax line: (302) 886-1528, Attention ZACTIMA ISS Safety Representative

To report to the local IRB, please follow SAE guidelines as outlined by the Office for the Dana Farber/Harvard Cancer Center Protection of Research Subjects (OPRS). Guidelines can be found on their website: .

If a non-serious AE becomes serious, this and other relevant follow-up information must also be provided to AstraZeneca, the FDA and Dana Farber / Harvard Cancer Center OPRS.

Serious adverse events that do not require expedited reporting to the FDA need to be reported to AstraZeneca preferably using the MedDRA coding language for serious adverse events. This information should be reported on a monthly basis and under no circumstance less frequently than quarterly.

All SAEs have to be reported to AstraZeneca, whether or not considered causally related to the investigational product. All SAEs will be documented.

8.3.2 Annual IND Report

In accordance with the regulation 21 CFR § 312.32, the Sponsor-Investigator shall within 60 days of the anniversary date that the IND went into effect submit a brief report of the progress of the investigation. Please refer to Code of Federal Regulations, 21 CFR § 312.32 for a list of the elements required for the annual report. All IND annual reports submitted to the FDA by the Sponsor-Investigator should be copied to Astra Zeneca.

8.3.3 Study Records

Study data will be collected in a standardized case report form (CRF). The investigator will record all patient information, including patient identification, tumor status, previous treatment, as well as information concerning drug administration, results of laboratory tests, toxicity and efficacy data.

9 STUDY CALENDAR

9.1 Pre-registration work-up

Informed consent must be obtained prior to performing any tests or procedures that are being done specific for the enrollment to this study. Medical history; clinical examination; ECOG PS; vital signs will be performed no sooner than 4 weeks prior to start of therapy; hematology; biochemistry; EKG will be performed within 7 days of initiation of protocol therapy. Appropriate radiographic procedures to document

extent of disease will be performed within 4 weeks prior to initiation of protocol therapy. Follow-up tumor assessments must be performed using the same method for a given patient (e.g. CT scan, Magnetic Resonance Imaging, X-rays, ultrasounds, scintigraphy, endoscopy). Ultrasound examination should only be used to detect new lesions or confirm subcutaneous skin lesion sizes. All concomitant medications will also be documented

9.2 Evaluation during treatment

Clinical examination including a complete physical examination, ECOG PS, vital signs, hematology, biochemistries, toxicity assessment and documentation of concomitant medications will be performed as noted in below required data table (Section 10). Toxicity will be assessed according to the NCI CTCAE V 3.0.

Initial tumor response will be assessed every 8 weeks and tumor assessment should be repeated no sooner than 4 weeks after a response for confirmation.

9.3 Post-treatment follow-up

After the end of study treatment (whatever the reason for discontinuation), the patient will be followed for at least one month, during which time all procedures for the reporting of SAEs will be followed. All patients who discontinue the trial secondary to an adverse event should be followed until resolution, stabilization or return to a baseline condition. Patients who discontinue the trial without documented tumor progression should be evaluated for extent of disease at the time of trial discontinuation.

10. REQUIRED DATA

Cycle	Screen *	Cycle 1				Cycle 2				Subsequent Cycles ***		Study Withdrawal
Week	0	1	2	3	4	1	2	3	4	1	3	
Informed consent	X											
Medical history	X											
Previous treatments (specific surgery, radiation, chemo)	X											
Inclusion/exclusion criteria	X											
Collect Tumor Blocks [A]	X											
Physical examination	X	X	X	X		X		X		X	X	X
Vital signs	X	X	X	X	X	X	X	X	X	X	X	
EKG	X †	X ‡	X		X				X	C		X
CBC with differential	X			X		X		X		X	X	X
Electrolytes (including K+, Ca++ and Mg++), creatinine, BUN, LFTs	X		X	X	X	X	X	X	X	X	X	
Urinalysis	X					X				X		
Pregnancy test **	X											
Performance Status	X	X		X		X		X		X	X	X
Imaging for RECIST measurements	X									X		X
Blood samples – plasma VEGF and circulating endothelial cells [V]		X[v]	X[v]	X[v]	X[v]					X[v]		
Tolerability/AE reporting	X	X	X	X		X		X		X	X	X
Assessment of Concomitant medication	X	X								X		

* within 4 wks prior to 1st dose of therapy, except lab work which must be within 7 working days prior to 1st dose of therapy.

** Females only; women who are documented to be postmenopausal for > 1 year or s/p hysterectomy do not need testing. Urine or serum

*** Lab work not needed on cetuximab-only days during subsequent cycles

† The screening QTc must be <480 msec or ≤460msec if patient is taking drugs known to cause Torsades de Pointes (Appendix A; Drug List 1, section 5.2) Up to 3 ECGs **may** be obtained at screening, and the mean QTc value used to determine eligibility.

‡ Within 3 days of or on C1 D1, "baseline" 12-lead ECGs are to be performed pre-dose. Baseline QTc will be determined by the average of no less than 3 consecutive ECGs (within 5-10 minutes of one another). Patients who are receiving drugs that may induce Torsades de Pointes must have an additional ECG obtained 4-8 hours after the first dose of ZD6474.

C After cycle 2, EKG required at week 12 (cycle 3, week 4)

[A] To be obtained from all patients on study – block does not need to arrive prior to starting therapy – only request to pathology made.

[V] See Appendix C – only for patients treated at MTD. Pre-treatment on cycle 1, day 1 and anytime on cycle 1, days 8, 15 and 22 and on day 1 of each subsequent cycle.

11 PATIENT WITHDRAWAL AND STUDY TERMINATION

Patients may withdraw from the trial at any time and for any reason. Some possible reasons for early withdrawal include the following:

- Disease progression
- Development of an intercurrent medical condition or need for concomitant treatment that precludes further participation in the trial
- Unacceptable toxicity or any adverse event (as outlined in section 7) that precludes further participation in the trial
- The investigator removes the patient from the trial in the best interests of the patient
- Inability of subject to comply with study requirements
- Determination by the investigator that it is no longer safe for the subject to continue therapy
- Study completion or discontinuation
- Patient withdraws consent to continued participation in the trial
- Interruption of treatment beyond allowed based on section 7

The reason and date of discontinuation are to be documented in the patient's medical record and in the CRF.

The investigator should complete all end of treatment procedures when a patient withdraws from treatment. All patients who discontinue the trial secondary to an adverse event should be followed until resolution, stabilization or return to a baseline condition.

ALL PATIENTS WHO RECEIVE ONE DOSE OF TREATMENT SHOULD BE INCLUDED IN ANY SAFETY ANALYSIS.

THE INVESTIGATOR MAY DISCONTINUE THE TRIAL AT ANY TIME. REASONS FOR EARLY TRIAL DISCONTINUATION MAY INCLUDE, BUT ARE NOT LIMITED TO, UNACCEPTABLE TOXICITY OF STUDY TREATMENT, A REQUEST TO DISCONTINUE THE TRIAL FROM A REGULATORY AUTHORITY OR AN IRB, OR POOR ENROLLMENT.

12 MEASUREMENT OF EFFECT

Response and progression will be evaluated in this study using the new international criteria proposed by the Response Evaluation Criteria in Solid Tumors (RECIST) Committee. Changes in only the largest diameter (unidimensional measurement) of the tumor lesions are used in the RECIST criteria. Note: Lesions are either measurable or non-measurable using the criteria provided below. The term

“evaluable” in reference to measurability will not be used because it does not provide additional meaning or accuracy.

12.1 Eligibility

Though the primary endpoint is determination of an MTD, the secondary endpoint of efficacy is very important in this study and patients must have measurable disease by RECIST.

Measurable disease - the presence of at least one measurable lesion. If the measurable disease is restricted to a solitary lesion, its neoplastic nature should be confirmed by cytology/histology.

Measurable lesions - lesions that can be accurately measured in at least one dimension with longest diameter $\geq$20 mm using conventional techniques or $\geq$10 mm with spiral CT scan.

Non-measurable lesions - all other lesions, including small lesions (longest diameter <20 mm with conventional techniques or <10 mm with spiral CT scan), i.e., bone lesions, leptomeningeal disease, ascites, pleural/pericardial effusion, inflammatory breast disease, lymphangitis cutis/pulmonis, cystic lesions, and also abdominal masses that are not confirmed and followed by imaging techniques.

All measurements should be taken and recorded in metric notation, using a ruler or calipers. All baseline evaluations should be performed as closely as possible to the beginning of treatment and never more than 4 weeks before the beginning of the treatment.

The same method of assessment and the same technique should be used to characterize each identified and reported lesion at baseline and during follow-up. Clinical lesions will only be considered measurable when they are superficial (e.g., skin nodules and palpable lymph nodes). For the case of skin lesions, documentation by color photography, including a ruler to estimate the size of the lesion, is recommended.

12.2 Methods of Measurement

CT and MRI are the best currently available and reproducible methods to measure target lesions selected for response assessment. The utilization of endoscopy and laparoscopy for objective tumor evaluation has not yet been fully and widely validated. Their uses in this specific context require sophisticated equipment and a high level of expertise that may only be available in some centers. Therefore, the utilization of such techniques for objective tumor response should be restricted to validation purposes in specialized centers. However, such techniques can be useful in confirming complete pathological response when biopsies are obtained.

Tumor markers alone cannot be used to assess response. If markers are initially above the upper normal limit, they must normalize for a patient to be considered in complete clinical response when all lesions have disappeared.

Cytology and histology can be used to differentiate between PR and CR in rare cases (e.g., after treatment to differentiate between residual benign lesions and residual malignant lesions in tumor types such as germ cell tumors).

12.3 Baseline documentation of "Target" and "Non-Target" lesions

All measurable lesions up to a maximum of five lesions per organ and 10 lesions in total, representative of all involved organs should be identified as ***target lesions*** and recorded and measured at baseline.

Target lesions should be selected on the basis of their size (lesions with the longest diameter) and their suitability for accurate repeated measurements (either by imaging techniques or clinically).

A sum of the longest diameter (LD) for *all target lesions* will be calculated and reported as the baseline sum LD. The baseline sum LD will be used as reference by which to characterize the objective tumor.

All other lesions (or sites of disease) should be identified as ***non-target lesions*** and should also be recorded at baseline. Measurements of these lesions are not required, but the presence or absence of each should be noted throughout follow-up.

12.4 Response Criteria

	**Evaluation of target lesions**
* Complete Response (CR):	Disappearance of all target lesions
* Partial Response (PR):	At least a 30% decrease in the sum of the LD of target lesions, taking as reference the baseline sum LD
* Progressive Disease (PD):	At least a 20% increase in the sum of the LD of target lesions, taking as reference the smallest sum LD recorded since the treatment started or the appearance of one or more new lesions
* Stable Disease (SD):	Neither sufficient shrinkage to qualify for PR nor sufficient increase to qualify for PD, taking as reference the smallest sum LD since the treatment started

	**Evaluation of non-target lesions**
* Complete Response (CR):	Disappearance of all non-target lesions and normalization of tumor marker level
* Incomplete Response/ Stable Disease (SD):	Persistence of one or more non-target lesion(s) or/and maintenance of tumor marker level above the normal limits
* Progressive Disease (PD):	Appearance of one or more new lesions and/or unequivocal progression of existing non-target lesions (1)

(1) Although a clear progression of "non target" lesions only is exceptional, in such circumstances, the opinion of the treating physician should prevail and the progression status should be confirmed later on by the review panel (or study chair).

12.5 Evaluation of best overall response

The best overall response is the best response recorded from the start of the treatment until disease progression/recurrence (taking as reference for PD the smallest measurements recorded since the treatment started). In general, the patient's best response assignment will depend on the achievement of both measurement and confirmation criteria

**Target lesions**	**Non-Target lesions**	**Evaluation of non-target lesions**	**Overall response**
CR	CR	No	CR
CR	Incomplete response/SD	No	PR
PR	Non-PD	No	PR
SD	Non-PD	No	SD
PD	Any	Yes or No	PD
Any	PD	Yes or No	PD
Any	Any	Yes	PD

Patients with a global deterioration of health status requiring discontinuation of treatment without objective evidence of disease progression at that time should be classified as having "symptomatic deterioration". Every effort should be made to document the objective progression even after discontinuation of treatment.
In some circumstances it may be difficult to distinguish residual disease from normal tissue. When the evaluation of complete response depends on this determination, it is recommended that the residual lesion be investigated (fine needle aspirate/biopsy) to confirm the complete response status.

12.6 Confirmation

The main goal of confirmation of objective response is to avoid overestimating the response rate observed. In cases where confirmation of response is not feasible, it should be made clear when reporting the outcome of such studies that the responses are not confirmed.

To be assigned a status of PR or CR, changes in tumor measurements must be confirmed by repeat assessments that should be performed no less than 4 weeks after the criteria for response are first met. Longer intervals as determined by the study protocol may also be appropriate. If a confirmation evaluation is obtained prior to the next required evaluation as described in section 10, the next evaluation should be

8 weeks after that scan and every 8 weeks thereafter. For example, if a patient has a PR and a confirmation scan is obtained at 4 weeks, the next scan should be 8 weeks after that.

In the case of SD, follow-up measurements must have met the SD criteria at least once after study entry at a minimum interval (in general, not less than 6-8 weeks) that is defined in the study protocol

12.7 Duration of overall response

The duration of overall response is measured from the time measurement criteria are met for CR or PR (whichever status is recorded first) until the first date that recurrence or PD is objectively documented, taking as reference for PD the smallest measurements recorded since the treatment started.

12.8 Reporting of results

All patients included in the study must be assessed for response to treatment, even if there are major protocol treatment deviations or if they are ineligible. Each patient will be assigned one of the following categories: 1) complete response, 2) partial response, 3) stable disease, 4) progressive disease, 5) early death from malignant disease, 6) early death from toxicity, 7) early death because of other cause, or 9) unknown (not assessable, insufficient data). All conclusions should be based on all eligible patients. Since patients will be treated with differing doses, response rate will be reported in descriptive analyses. Since this is a phase I study, response rates for each dose level and the MTD will be reported and considered secondary analyses.

Exploratory subanalyses may then be performed on the basis of a subset of patients, excluding those for whom major protocol deviations have been identified (e.g., early death due to other reasons, early discontinuation of treatment, major protocol violations, etc.). However, these subanalyses may not serve as the basis for drawing conclusions concerning treatment efficacy, and the reasons for excluding patients from the analysis should be clearly reported.

13 Statistical Considerations

This is a phase I study, initially with dose escalation of ZD6474 and then adding irinotecan. The study will likely accrue between 6 and 15 patients (before MTD testing) with the described dose escalation schema, though it could range from 3 to 34 patients. We expect accrual to be 2-3 patients / month (though this will vary during waiting periods to proceed to next dose level). If patients in dose levels 2-4 who have a DLT in the first 14 days (prior to initiation of irinotecan), the patient will not be counted towards the DLTs of that cohort and an additional patient will be substituted. For example, if one of the first 3 patients in dose level 4 have a DLT, that patient will not be calculated into the DLT tabulation for ZD6474,

cetuximab and irinotecan and an additional patient will be substitute in that spot. If this scenario occurs twice, the study will be held and the study team will reevaluate the ZD6474 / cetuximab toxicity profile. To ensure the toxicity at the MTD is acceptable and to further define any toxicity that may occur with this regimen, an additional 10 patients will be accrued at the MTD. The estimation of toxicity rate will be based on these 10 additional patients. With 10 patients, the 90% confidence bounds for any toxicity will be +/- 28%.The probability of a dose level declared too toxic based on the true DLT rate is defined as such:

True DLT Rate	Probability of declaring dose too toxic
20%	0.29
30%	0.51
40%	0.69
50%	0.83
60%	0.92

Response rate, progression-free survival and overall survival are secondary analyses. Each will be reported by dose level and at the MTD.

14 Ethical and Regulatory Considerations

14.1 Ethical principles

The study should be conducted according to the principles outlined by the 18th World Medical Assembly (Helsinki, 1964) and all applicable amendments; the International Conference on Harmonization Guidelines for Good Clinical Practice; and FDA regulations regarding the conduct of clinical trials and the protection of human subjects.

14.2 Informed consent

It is the responsibility of the Investigator to obtain written informed consent from a patient or a patient's legal representative before any study related procedures are performed. The Investigator will provide an informed consent in compliance with ICH GCP and U.S. FDA guidelines (21 CFR 50). The informed consent document must clearly describe the potential risks and benefits of the trial, and each prospective participant must be given adequate time to discuss the trial with the Investigator or site staff and to decide whether or not to participate. The informed consent must be approved by the IRB prior to being presented to a potential patient.

One copy of the patient's signed, dated and witnessed written consent will be kept in the patient's medical record and one copy will be given to the patient or the patient's legal representative.

14.3 Institutional Review Board (IRB) Approval

The Investigator must obtain the approval of the protocol, the informed consent document and any other material used to inform the patient about the nature of the trial from the local IRB in the form of a written letter. On the approval letter, the following items should be clearly stated: trial title, protocol number and version, study-related documents (protocol, informed consent material, advertisement when applicable), IRB review date, and IRB decision. The trial should not start until a copy of this written approval has been received by the Investigator.

The Principal Investigator is responsible for keeping the IRB apprised of the progress of the study and of any changes made to the protocol as deemed appropriate, but in any case the IRB must be updated at least once a year. The Principal Investigator must also keep the IRB informed of any significant adverse events.

Investigators are required to promptly notify their respective IRB of all adverse drug reactions that are both serious and unexpected. This generally refers to serious adverse events that are not already identified in the Investigator Brochure and that are considered possibly or probably related to the study drug by the investigator. Investigators must immediately forward to their IRB any written safety report or update provided by Astra-Zeneca (e.g., IND safety report, Investigator Brochure, safety amendments and updates, etc.).

14.4 Additional Responsibilities of the Investigator

The Investigator(s) agrees to perform the study in accordance with ICH Good Clinical Practice and FDA regulations. The Investigator is required to ensure compliance with respect to the investigational drug schedule, visit schedule and procedures required by the protocol.

The Investigator should be able to recruit the required number of suitable patients and should have sufficient time to properly conduct and complete the trial. The Investigator should have available an adequate number of qualified staff and adequate facilities for the duration of the trial, and should ensure that all persons assisting with the trial are adequately informed about the protocol, the protocol-defined procedures, protocol therapy and trial related duties and functions..

The Investigator should be responsible for all trial-related medical decisions. During and following a patient's participation in a trial, the investigator should ensure that adequate medical care is provided to a patient for any adverse events related to the trial.

14.5 Confidentiality

It is the responsibility of the Investigator to ensure that the confidentiality of all patients participating in the trial and all of their medical information is maintained. All case report forms and any identifying information must be kept in a secure location with access limited to the study staff directly participating in the trial. Personal medical information may be reviewed by representative (s) of Astra Zeneca, the IRB or of regulatory authorities in the course of auditing the trial. Every reasonable effort will be made to maintain such information as confidential.

14.6 Protocol Amendment

Any changes to this protocol made by the Investigator must be in the form of a written amendment and the amendment will be appended to this protocol.

Approval of amendments by the IRB is required prior to their implementation, unless there are overriding safety reasons. If the change or deviation increases risk to the study population, or adversely affects the validity of the clinical investigation or the subject's rights, full approval must be obtained prior to implementation. For changes that do not involve increased risk or affect the validity of the investigation or the subject's rights, approval may be obtained by expedited review. When appropriate, an amendment may require a change to a written consent form as well. All changes will also be forwarded to Astra Zeneca.

14.7 Audits

Authorized representatives of AstraZeneca, a regulatory authority, an Independent Ethics Committee (IEC) or an Institutional Review Board (IRB) may visit the centre to perform audits or inspections, including source data verification. The purpose of an AstraZeneca audit or inspection is to systematically and independently examine all study-related activities and documents to determine whether these activities were conducted, and data were recorded, analyzed, and accurately reported according to the protocol, Good Clinical Practice (GCP), guidelines of the International Conference on Harmonization (ICH), and any applicable regulatory requirements. The investigator should contact AstraZeneca immediately if contacted by a regulatory agency about an inspection at his center.

14.8 Patient Data Protection

Federal law requires Dana Farber/Harvard Cancer Center (DF/HCC) and its affiliated research doctors, health care providers, and physician network to protect the privacy of information that identifies patients and relates to your past, present, and future physical and mental health conditions (“protected health information”). The written informed consent form will explain that study data will be stored in a computer database, maintaining confidentiality in accordance with national data legislation. A detailed explanation about what data will be used or shared for research purposes, methods to protect information, whom data can be shared with

(including individuals or entities to perform functions on behalf of DF/HCC and its affiliates, the sponsor of the trial AstraZeneca, and federal and state agencies, data safety monitoring boards), and statement of privacy.

References:

1. Jemal A, Murray T, Ward E, et al: Cancer statistics, 2005. CA Cancer J Clin 55:10-30, 2005

2. Scheithauer W, Rosen H, Kornek GV, et al: Randomised comparison of combination chemotherapy plus supportive care with supportive care alone in patients with metastatic colorectal cancer. BMJ 306:752-5, 1993

3. Smyth JF, Hardcastle JD, Denton G, et al: Two phase III trials of tauromustine (TCNU) in advanced colorectal cancer. Ann Oncol 6:948-9, 1995

4. Cunningham D, Pyrhonen S, James RD, et al: Randomised trial of irinotecan plus supportive care versus supportive care alone after fluorouracil failure for patients with metastatic colorectal cancer. Lancet 352:1413-8, 1998

5. Allen-Mersh TG, Earlam S, Fordy C, et al: Quality of life and survival with continuous hepatic-artery floxuridine infusion for colorectal liver metastases. Lancet 344:1255-60, 1994

6. Meyerhardt JA, Mayer RJ: Systemic therapy for colorectal cancer. N Engl J Med 352:476-87, 2005

7. Baselga J, Albanell J: Epithelial growth factor receptor interacting agents. Hematol Oncol Clin North Am 16:1041-63, 2002

8. Mayer A, Takimoto M, Fritz E, et al: The prognostic significance of proliferating cell nuclear antigen, epidermal growth factor receptor, and mdr gene expression in colorectal cancer. Cancer 71:2454-60, 1993

9. Saltz L, Rubin M, Hochster H, et al: Cetuximab (IMC-C225) Plus Irinotecan (CPT-11) is Active in CPT-11-Refractory Colorectal Cancer (CRC) that Expresses Epidermal Growth Factor Receptor (EGFR). Proc Am Soc Clin Oncol 20:3a, 2001

10. Saltz LB, Meropol NJ, Loehrer PJ, Sr., et al: Phase II trial of cetuximab in patients with refractory colorectal cancer that expresses the epidermal growth factor receptor. J Clin Oncol 22:1201-8, 2004

11. Cunningham D, Humblet Y, Siena S, et al: Cetuximab (C225) alone or in combination with irinotecan (CPT-11) in patients with epidermal growth factor receptor (EGFR)-positive, irinotecan-refractory metastatic colorectal cancer (MCRC). N Engl J Med 351:337-345., 2004

12. Folkman J: Tumor angiogenesis: therapeutic implications. N Engl J Med 285:1182-6, 1971

13. Ferrara N, Gerber HP, LeCouter J: The biology of VEGF and its receptors. Nat Med 9:669-76, 2003

14. Berlin JD: Targeting vascular endothelial growth factor in colorectal cancer. Oncology (Huntingt) 16:13-5, 2002

15. Kabbinavar F, Hurwitz HI, Fehrenbacher L, et al: Phase II, randomized trial comparing bevacizumab plus fluorouracil (FU)/leucovorin (LV) with FU/LV alone in patients with metastatic colorectal cancer. J Clin Oncol 21:60-5, 2003

16. Hurwitz H, Fehrenbacher L, Novotny W, et al: Bevacizumab plus irinotecan, fluorouracil, and leucovorin for metastatic colorectal cancer. N Engl J Med 350:2335-42, 2004

17. Matar P, Rojo F, Cassia R, et al: Combined epidermal growth factor receptor targeting with the tyrosine kinase inhibitor gefitinib (ZD1839) and the monoclonal antibody cetuximab (IMC-C225): superiority over single-agent receptor targeting. Clin Cancer Res 10:6487-501, 2004

18. Huang S, Armstrong EA, Benavente S, et al: Dual-agent molecular targeting of the epidermal growth factor receptor (EGFR): combining anti-EGFR antibody with tyrosine kinase inhibitor. Cancer Res 64:5355-62, 2004

19. Wedge SR, Ogilvie DJ, Dukes M, et al: ZD6474 inhibits vascular endothelial growth factor signaling, angiogenesis, and tumor growth following oral administration. Cancer Res 62:4645-55, 2002

20. Carlomagno F, Vitagliano D, Guida T, et al: ZD6474, an orally available inhibitor of KDR tyrosine kinase activity, efficiently blocks oncogenic RET kinases. Cancer Res 62:7284-90, 2002

21. Ogino S, Meyerhardt JA, Cantor M, et al: Molecular alterations in tumors and response to combination chemotherapy with gefitinib for advanced colorectal cancer. Clin Cancer Res 11:6650-6, 2005

22. Ogino S, Kawasaki T, Brahmandam M, et al: Sensitive sequencing method for KRAS mutation detection by Pyrosequencing. J Mol Diagn 7:413-21, 2005

23. Cianchi F, Cortesini C, Fantappie O, et al: Inducible nitric oxide synthase expression in human colorectal cancer: correlation with tumor angiogenesis. Am J Pathol 162:793-801, 2003

Appendix A: Medications Known To Prolong The Interval and/or Induce Torsades De Pointes (Tdp)

It has been recognized for a number of years that certain prescription medications can prolong the QT/QTc interval and cause a form of acquired Long QT syndrome, known as drug induced LQTS.

For patients taking medications with known risk of Torsades de Pointes:

- Screening QTc must be ≤460 msec
- An additional ECG must be obtained 4-8 hours after the first dose of ZD6474

The drugs that prolong the QT interval and/or have a risk of inducing Torsade de Pointes (TdP) are listed below. We have divided these into two groups based on their known or perceived risk of causing TdP:

Group 1. Drugs That are Generally Accepted by Authorities to Have a Risk of Causing Torsades de Pointes

Table 1 **Group 1 Drugs**		
Drug (Generic Names)	Drug Class (Clinical Usage)	Comments
Albuterol (by parenteral administration)	Bronchodilator (asthma)	Inhaled Albuterol at normal doses acceptable
Amiodarone	Anti-arrhythmic (heart rhythm)	F>M, TdP Cases in Literature
Arsenic trioxide	Anti-cancer (leukaemia)	TdP Cases in Literature
Bepridil	Anti-anginal (heart pain)	F>M
Chlorpromazine	Anti-psychotic/antiemetic (schizophrenia/nausea)	TdP Cases in Literature
Chloroquine	Anti-malaria (malaria infection)	
Cisapride	GI stimulant (stimulates GI motility)	Open Prescription Restricted F>M
Disopyramide	Anti-arrhythmic (heart rhythm)	F>M
Dofetilide	Anti-arrhythmic (heart rhythm)	
Domperidone	Anti-nausea (nausea)	
Droperidol	Sedative/hypnotic (anaesthesia adjunct)	TdP Cases in Literature
Erythromycin	Antibiotic/GI stimulant (infection/GI motility)	F>M
Halofantrine	Anti-malarial (malaria infection)	F>M
Haloperidol	Anti-psychotic	

Table 1 **Group 1 Drugs**		
Drug (Generic Names)	Drug Class (Clinical Usage)	Comments
	(schizophrenia, agitation)	
Ibutilide	Anti-arrhythmic (heart rhythm)	F>M
Levomethadyl	Opiate agonist (narcotic dependence)	
Mesoridazine	Anti-psychotic (schizophrenia)	
Methadone	Opiate agonist (pain control/ narcotic dependence)	F>M
Pentamidine	Anti-infective (pneumocystis pneumonia)	F>M
Pimozide	Anti-psychotic (Tourette's tics)	F>M, TdP Cases in Literature
Procainamide	Anti-arrhythmic (heart rhythm)	
Quinidine	Anti-arrhythmic (abnormal heart rhythm)	F>M
Salbutamol (by parenteral administration)	Bronchodilator (asthma)	Inhaled salbutamol at normal doses acceptable
Sotalol	Anti-arrhythmic (heart rhythm)	F>M
Sparfloxacin	Antibiotic (bacterial infection)	
Thioridazine	Anti-psychotic (schizophrenia)	

Group 2. Drugs That in Some Reports May be Associated With Torsades de Pointes But at This Time Lack Substantial Evidence of Causing Torsades de Pointes

Table 2 **Group 2 Drugs**		
Drug (Brand Names)	Drug Class (Clinical Usage)	Comments
Alfuzocin	Alpha 1-blocker (Benign prostatic hyperplasia)	
Amantadine	Dopaminergic/Anti-viral/Anti-infective (Parkinson’s disease)	
Amitriptyline	Tricyclic anti-depressant (depression)	
Amoxapine	Tricyclic anti-depressant (depression)	
Azithromycin	Antibiotic (bacterial infection)	

Table 2 **Group 2 Drugs**		
Drug (Brand Names)	Drug Class (Clinical Usage)	Comments
Citalopram	Anti-depressant (depression)	
Clarithromycin	Antibiotic (bacterial infection)	TdP Cases in Literature
Clomipramine	Tricyclic antidepressant (depression)	
Chloral hydrate	Sedative (sedation/insomnia)	
Clozapine	Anti-psychotic (schizophrenia)	
Desipramine	Tricyclic anti-depressant (depression)	TdP Cases in Literature
Dolastron	Anti-nausea (nausea and vomiting)	
Doxepin	Anti-depressant (depression)	TdP Cases in Literature
Felbamate	Anti-convulsant (seizures)	
Flecainide	Anti-arrhythmic (heart rhythm)	Association not clear
Fluconazole	Anti-fungal (fungal infection)	
Fluoxetine	Anti-depressant (depression)	Association not clear
Foscarnet	Antiviral (HIV infection)	
Fosphenytoin	Anticonvulsant (seizures)	
Gatifloxacin	Antibiotic (bacterial infection)	
Gemifloxacin	Antibiotic (bacterial infection)	
Granisetron	Anti-nausea (nausea and vomiting)	
Imipramine	Anti-depressant (depression, pain, other)	TdP Cases in Literature
Indapamide	Diuretic (stimulates urine & salt loss)	TdP Cases in Literature, QT in animals
Isradipine	Anti-hypertensive (high blood pressure)	
Levofloxacin	Antibiotic (bacterial infection)	Association not clear
Lithium	Anti-mania (bipolar disorder)	
Mexilitine	Anti-arrhythmic (abnormal heart rhythm)	
Moexipril/HCTZ	Anti-hypertensive (high blood pressure)	
Moxifloxacin	Antibiotic (bacterial infection)	

Table 2 **Group 2 Drugs**		
Drug (Brand Names)	Drug Class (Clinical Usage)	Comments
Nicardipine	Anti-hypertensive (high blood pressure)	
Nortriptyline	Tricyclic antidepressant (depression)	
Octreotide	Endocrine (acromegaly/carcinoid diarrhoea)	
Ofloxacin		
Ondansetron	Anti-emetic (nausea and vomiting)	
Paroxetine	Anti-depressant (depression)	
Protriptyline	Tricyclic antidepressant (depression)	
Quetiapine	Anti-psychotic (schizophrenia)	
Risperidone	Anti-psychotic (schizophrenia)	
Roxithromycin	Antibiotic (bacterial infection)	
Salmeterol	Sympathomimetic (asthma, COPD)	
Sertraline	Antidepressant (depression)	Association not clear
Solifenacin	Muscarinic receptor antagonist (treatment of overactive bladder)	
Tacrolimus	Immune suppressant	TdP Cases in Literature
Tamoxifen	Anti-cancer (breast cancer)	
Telithromycin	Antibiotic (bacterial infection)	
Tizanidine	Muscle relaxant	
Trimipramine	Tricyclic antidepressant (depression)	
Vardenafil	Phosphodiesterase inhibitor (vasodilator)	
Venlafaxine	Antidepressant (depression)	
Voriconazole	Anti-fungal (fungal infection)	
Ziprasidone	Anti-psychotic (schizophrenia)	

Appendix B: New York Heart Association (NYHA) Cardiac Classification

The NYHA classification system relates symptoms to everyday activities and the patient's quality of life.

Class	Symptoms
Class I (Mild)	No limitation of physical activity. Ordinary physical activity does not cause undue fatigue, palpitation, or dyspnea (shortness of breath).
Class II (Mild)	Slight limitation of physical activity. Comfortable at rest, but ordinary physical activity results in fatigue, palpitation, or dyspnea.
Class III (Moderate)	Marked limitation of physical activity. Comfortable at rest, but less than ordinary activity causes fatigue, palpitation, or dyspnea.
Class IV (Severe)	Unable to carry out any physical activity without discomfort. Symptoms of cardiac insufficiency at rest. If any physical activity is undertaken, discomfort is increased.

APPENDIX C: Correlative Science

1. General

The purpose of the correlative science studies is to explore if there are potential signals that may be predictive of activity of combined EGFR and VEGF inhibition. Samples will be based on prior archived biopsies (for patients that have both a biopsy from the primary tumor and site of metastases, both samples will be used) and serum samples at intermittent time points. The samples will not be used for genetic testing or for the generation of immortalized cell lines. Tumor blocks will be obtained from all patients in DLT finding phase and MTD phase of trial. Serum sample will only be drawn and required from patients treating during MTD phase.

The analyses of tumor blocks will be performed in the laboratory of Drs. Shuji Ogino and Charles Fuchs. The analyses of plasma markers and circulating endothelial cells were be performed in the laboratory of Dr. Rakesh Jain.

Upon receiving the samples they will be re-labeled with a unique identifier. The code key will be kept in a password protected program only available to investigators in the clinical trial.

Tissue collection will consist of uncut paraffin embedded blocks that currently exist either in DFHCC institutions or in referring institutions. If a biopsy or resection has been performed at a non-DFHCC institution, tissue blocks will be requested from the referring institution. In this situation, a copy of the patient's informed consent form will be provided to the referring institution. Alternatively, if the pathology department at the referring institution will provide only unstained sections, 20 slides of 5 micron thickness. Any remaining samples and test samples, such as unstained slides will be retained at the testing laboratory until completion of the trial. All samples will be destroyed after a maximum of 10 years.

Serum samples will be drawn by an experienced phlebotomist or a research nurse as specific below.

It is not proposed that the analysis of results from a specific subject are made available routinely to either the subject or the treating physician, however, group results from this trial will be published.

2. Proposed markers of tumor tissue to test

We will explore molecular alterations in tumor blocks in VEGF, CD31 (for microvessel density), EGFR, p21, p53, p-AKT1, MSI, Kras, and BRAF. In a recent report by our group of patients treated with gefitinib, irinotecan, 5-FU and leucovorin as initial chemotherapy for metastatic colorectal cancer, patients with tumors demonstrating p21 expression, combined p21 and p53 expression, and p-AKT1 expression (downstream

effectors of EGFR) had a lower likelihood of having a radiographic response to therapy.[21] Mutations in *K-ras* may predict resistance to gefitinib in lung cancer.

We will also explore plasma VEGF and other angiogenesis cytokine levels (see below) prior to treatment and at 3 times points after treatment commences. Blood samples will be collected on day 1 prior to the start of therapy, on day 8, on day 22 and at week 9.

3. Methods for tumor tissue testing

PCR and Sequencing KRAS and BRAF. Tumor tissue (and normal tissue if applicable) will be reviewed and dissected from tissue sections obtained from the paraffin tissue blocks, and genomic DNA will be extracted using QIAmp DNA Mini Kit. For whole genome amplification (WGA), genomic DNA will be PCR-amplified using random 15-mer primers. *KRAS* and *BRAF* sequencing will be performed using Pyrosequencing technology as previously described by Ogino et al.[22]

Microsatellite Instability (MSI). We determine MSI status by PCR of BAT25, BAT26 and BAT40 microsatellites as previously described.[22] Tumors with either unstable BAT25 or BAT26 will be diagnosed as MSI-high. If no normal tissue is available, tumors that show two peaks in at least two microsatellites will be diagnosed as MSI-high.

Immunohistochemical Analyses. For EGFR immunohistochemistry, paraffin sections of CRC will be deparaffinized, incubated with 3% H_2O_2 (20 min) to block endogenous peroxidase, and then incubated with pepsin at 37°C (10 min). Protein block (Vector Laboratories, Burlingame, CA) (20 min) will be followed by application of primary anti-EGFR antibody (Zymed Laboratories, South San Francisco, CA) (dilution 1:100; overnight at 4°C). Then, secondary anti-mouse antibody (Vector Laboratories) was applied (20 min), avidin biotin complex will be added and sections visualized by diaminobenzidine (DAB) (5 min) and methyl-green counterstain. EGFR expression will be recorded as negative (0), weakly positive (1+), positive (2+), or strongly positive (3+). The extent of staining will also be recorded.

For phosphorylated AKT1 (p-AKT1) immunohistochemistry, antigen retrieval will be performed by incubating deparaffinized tissue sections in citrate buffer by a microwave at 92.8°C for 15 min and 30 min. Tissue sections will be then incubated with 3% H_2O_2 (30 min) to block endogenous peroxidase, and then incubated with protein block (Vector Laboratories) (30 min). Primary anti-p-AKT1 antibody (Cell Signaling, Beverly, MA) (dilution 1:50) was applied for overnight at 4°C. Then, secondary anti-rabbit antibody (Vector Laboratories) will be applied (30 min), avidin biotin complex will be added and sections visualized by DAB (5 min) and methyl-green counterstain. p-AKT1 expression was interpreted as negative or positive, using normal epithelial cells and lymphocytes as reference.

For p21 (CDKN1A/CIP1) and p27 (CDKN1B/KIP1) immunohistochemistry, antigen retrieval will be performed by incubating deparaffinized tissue sections in citrate buffer at

high power in a microwave for 30 min (in a pressure cooker) and 15 min, respectively. Tissue sections will then incubated with 3% H_2O_2 (10 min) to block endogenous peroxidase, and then incubated with protein block (Vector Laboratories) (10 min). Primary anti-p21 antibody (Pharmingen, San Diego, CA) (dilution 1:50) or anti-p27 antibody (Transduction Laboratories, San Diego, CA) (dilution 1:200) will be applied for 30 min at room temperature. Then, biotinylated secondary Multi-Link antibody (Biogenex, San Ramon, CA) will be applied (20 min), horse radish peroxidase avidin complex (Biogenex) will be added and sections visualized by DAB (30 sec) and methyl-green counterstain. p21 expression will be recorded as negative (< 10% of cells staining) or positive (≥ 10% of cells staining). and p27 expression will be recorded as negative (≤ 20% of cells staining) or positive (> 20% of cells staining).

We will assess for mutations in p53 (*TP53*) by immunohistochemistry. Methods for p53 immunohistochemistry were previously described.[21] Only strong and unequivocal staining with ≥ 50% of cells staining will be interpreted as positive. Weak or staining in <50% of cells for p53 will be regarded as negative.

VEGF immunohistochemistry will be performed as previously described[23] with polyclonal anti-VEGF (A-20, Santa Cruz Biotechnology) will be used at 1:100 dilution. For evaluation of tumor microvessel density (MVD), we will use an automated imaging system, Ariol 2.0 (Applied Imaging), and CD31 immunohistochemistry that positively stains vascular endothelial cells. CD31 immunohistochemistry will be performed as previously described[23] with anti-CD31 (JC70; Dako), dilution 1:10. Individual microvessels will be counted in a single 250x field of a highly vascular tumor area excluding ulceration or granulation tissue. MVD in each tumor will be defined as the microvessel count of the field with the highest number of microvessels.

All immunohistochemical and molecular results will be interpreted as blinded from patients' identity and clinical outcomes data.

4. **Serum Marker Studies**

To measure numerous biomarkers simultaneously with exceptional sensitivity but only small amount (~10μl) of plasma proteins, the MSD (Meso Scale Discovery, Maryland) platform will be used. MSD technology utilizes electrochemiluminescence detection to detect binding events on patterned arrays. By customizing the Multi-Array, up to 10 plasma molecules can be simultaneously tested (Willett et al., JCO 2005). We will collect blood samples from the patients before and during the course of treatment (see below), and use MSD technology to measure tumstatin, as well as other well established biomarkers such as VEGF-A, –D, sVEGFRs, PlGF, EGF, TGF-α, PDGF-AA, PDGF-AB, PDGF-BB, angiopoietin-1, thrombospondin-1 and IL-8 . The level of these markers during treatment will be compared to the baseline level.

4.1 Sample Collection Time Points

Blood samples will be obtained for protein analysis of potential biomarkers for anti-angiogenic therapy at the following time points:

- Prior to starting therapy on day 1 of cycle 1
- Days +8, 15, 22
- Day 1 of start of every other cycle until patient goes off study

4.2 Supplies

Blood collection supplies (needed for each time point sample)

- Acceptable Tubes
  - SARSTEDT Monovette® EDTA KE (9 ml), Part # 02.1333.001 **or**
  - Becton-Dickinson Vacutainer™ K2E (10 ml), Part # 367525 **or**
  - Greiner Bio-One Vacuette® K3E EDTA K3 (9 ml), Part 455036
- Blood tubes must be gently inverted several times after collection to ensure thorough mixing of EDTA with the sample to prevent clotting.
- **Glass tubes MUST NOT be used** as they may break during transport
and freeze-thaw cycles.
- **Heparin must not be used as an** anticoagulant as it may interfere with downstream genotyping methodology.
- Ten (10) red-labeled Nalgene cryovials

4.3 Sample Labels

For each blood collection, prepare two red (for plasma) labels each printed with Study-No., patient ID, initials and day/time of sample collection (24-hour clock format, *i.e.*, 6:30 pm = 18:30). Alternatively, purple screw caps can be used to color code the vials.

For one timepoint per patient, label one tube as “do not discard” (for the purpose of extracting genomic DNA in the future)

4.4 Collection and Handling

Blood (Plasma) collection

- Collect ~ 10 ml of blood into any of the tubes listed 4.2. The tubes
must be pre-cooled in an ice bath.
- Gently invert five to six times to ensure adequate mixing and prevent
coagulation.
- Cool the tubes immediately in an ice bath.
- Centrifuge at 2000 RPM for 20 minutes at 4 degrees C without break
- Plasma is pipetted in ten4-5 ml aliquots and frozen at -80 °C until shipped (on dry ice)
- Save and freeze the red cell layer at -80 °C from one timepoint per patient (otherwise disregard red cell layer)

Shipment and Packaging of the Blood and Plasma Samples

For patients treated at MGH, the blood samples will be packaged in isothermal container maintained at +0°C with wet ice. The samples will be delivered within 2 hours after collection. The address is:

```
Sylvie Roberge / Christina Koppel
Steele Laboratory for Tumor Biology
Department of Radiation Oncology
Massechusettes General Hospital
100 Blossom Street, COX-734
Boston, MA 02114
Tel: (617) 726-8143
     (617) 724-1353
Fax: (617) 724-5841
Pager #14082
```

5. Circulating Endothelial Cells Assay

Targeting angiogenic vessels requires adequate methods for the assessment of the biologic effect of various new drugs developed to control cancer progression. Tumor angiogenesis is evaluated mainly by measuring microvessel density (MVD) in biopsy specimens using immunohistochemistry. Predicting and/or assessing accurately the efficacy of antiangiogenic therapies by this method is hampered by the heterogeneity of tumors, and by the difficulty to obtain specimens at multiple time points during treatments. On the other hand, the number of circulating endothelial cells (CECs) – measured by flow cytometry – is significantly increased in the peripheral blood of untreated lymphoma and breast cancer patients (Mancuso et al. 2001). Furthermore it has been shown that in lymphoma patients achieving complete remission after chemotherapy, the number of CECs was reduced to the values observed in healthy controls, and activated CECs were found to decrease in breast cancer patients evaluated before and after quadrantectomy. The strong correlation observed between the number of CECs and tumor volume indicates that the number of CECs increase with tumor progression (Monestiroli et al. 2001). The evaluation of CEC kinetics and viability before and after drug therapy demonstrated that in control animals, most CECs seem to have initiated an apoptotic program, whereas CEC viability is markedly improved in tumor-bearing mice. Moreover, a correlation has been observed between CEC and plasma levels of human VEGF (produced by tumor cells), suggesting that the antiapoptotic properties of VEGF may also play a role in determining the number of CECs in tumor-bearing mice. The increase in CECs has been attributed to shedding from tumor vessels or from distant sites and uninvolved vessels that are activated by cytokines produced by the tumor (such as from the bone marrow, Ref (Rafii et al. 2002). We have recently characterized the phenotype and kinetics of CECs in peripheral blood of rectal cancer patients during VEGF-blockade (Duda et al., JCO 2006).

We have recently shown in humans that this method may be used for evaluation of the early response to VEGF blockade in patients with rectal carcinoma (Willett et al. 2004 and 2005). Specifically, a decrease in both viable CECs and progenitor cell number was noted 3 days after administration of the VEGF-specific antibody, bevacizumab.

This decrease was sustained at day 12 (which is close to the half-life of bevacizumab in circulation) only in patients who received the high-dose of 10 mg/kg bevacizumab. This difference between the high- and low-dose of bevacizumab may have been caused by the significant increase in plasma VEGF and PlGF in all patients at both day 3 and day 12 compared to pre-treatment (Willett et al., 2005).

5.1 Sample Collection Time Points

Blood samples will be obtained for CEC analysis for anti-angiogenic therapy at one timepoint per patient.

Phase I Study of ZD6474, Cetuximab and Irinotecan in Patients with Metastatic Colorectal Cancer

Name: **Patient Signature:** **RN signature:**

Date **Date**

Cycle # Week #	MM/DD/YYYY	MM/DD/YYYY	MM/DD/YYYY	MM/DD/YYYY	MM/DD/YYYY	MM/DD/YYYY	MM/DD/YYYY
# of Tablets							
Dose							
Time Drug Taken							
Symptoms							
Cycle # Week #	MM/DD/YYYY	MM/DD/YYYY	MM/DD/YYYY	MM/DD/YYYY	MM/DD/YYYY	MM/DD/YYYY	MM/DD/YYYY
# of Tablets							
Dose							
Time Drug Taken							
Symptoms							
Cycle # Week #	MM/DD/YYYY	MM/DD/YYYY	MM/DD/YYYY	MM/DD/YYYY	MM/DD/YYYY	MM/DD/YYYY	MM/DD/YYYY
# of Tablets							
Dose							
Time Drug Taken							
Symptoms							
Cycle # Week #	MM/DD/YYYY	MM/DD/YYYY	MM/DD/YYYY	MM/DD/YYYY	MM/DD/YYYY	MM/DD/YYYY	MM/DD/YYYY
# of Tablets							
Dose							
Time Drug Taken							
Symptoms							
